# 

**Published:** 1877-12

**Authors:** 


### EDITORIAL.

### BOSTON JOURNAL OF CHEMISTRY.

### VIRGINIA STATE DENTAL ASSOCIATION.

The annual meeting of this body will be held in the city of Richmond, on Tuesday December 11th, 1877 at 10 o'clock A.M.

A full attendance is desirable as subjects of importance to the association as well as the profession at large will be discussed.

The following regular standing committees will report, viz:

*Comittee on Publication*—Dr. S. H. Henckel, Staunton, Va.; Dr. L. Sydnor, Salem, Va.; Dr. Frank Harris, Harrisonburg, Va.

*Committee on Operative Dentistry*—Dr. James F. Thompson, Fredericksburg, Va.; Dr. C. A. Mercer, Richmond, Va.; Dr. G. A. Sprinkel, Culpeper, Va.

*Committee on Mechanical Dentistry*—Dr. J. Hall Moore, Richmond, Va.; Dr. George Chewning, Fredricksburg, Va.; Dr. E. R. Perrow, Lovingston, Va.

*Committee on Dental Education and Literature*—Dr. James Johnston, Staunton, Va.; Dr. George F. Keesee, Richmond, Va.; Dr. G. Smith, Louisa Courthouse, Va.

*Committee on Dental Pathology*—Dr. George B. Steel, Richmond, Va.; Dr. J. W. Scribner, Charlottesville, Va.; Dr. A. C. Smith, Louisa Courthouse, Va.

*Committee on Physiology, Histology, Microscopy and Chemistry*—Dr. Judd, B. Wood, Richmond, Va.; Dr. W. Leigh Burton, Richmond, Va; Dr. F. A. Jeter, Richmond, Va.

*Executive Committee*—Dr. L. M. Cowardin, Richmond, Va.; Dr. F. A. Jeter, Richmond, Va.; Dr. F. L. Harris, Harrisonburg, Va.

W. W. H. THACKSTON, President.

J. W. SCRIBNER, D. D. S.,
Corresponding Secretary.

### CLOSE OF THE VOLUME.

With this number the thirty-first volume of the REGISTER is closed, and how quickly the time has passed since the end of the thirtieth volume. We trust that during the past year the journal has not fallen back, but we hope and think it has made progress; that a careful perusal of its pages, for this period will find a very considerable amount of valuable and interesting matter.

It is a matter of discouragement that so few read our professional journals even of those who take them.

Perhaps not more than one in ten of the profession take any dental journal and not more than one in five of those who do take them read them carefully and profitably. This is one of the discouragements that attaches to dental journalism. But notwithstanding all the discouragements we will proceed to the work of another year, with, we hope, better results than in the past. We shall hope for larger co-operation than in the past.

AMERICAN DENTAL ASSOCIATION—Meets on the first Tuesday in Aug., 1877, at Chicago. *Pres.* G. W. Keeley, Oxford, Ohio; *Cor. Sec.* J. H. McQuilien, Philadel'a; *Rec. Sec.* C. Stoddard Smith, Springfield, Ill.

*American Dental Convention* Meets at Deer Park, Md., second Tuesday in August, 1877. *Pres.* W H. Atkinson, New York; *Cor. Sec.* G. W. Mills, Baltimore; *Rec. Sec.* A. Tees, Philadelphia, Pa.

*List of Societies Represented in the American Dental Association.*

AMERICAN ACADEMY OF DENTAL SURGERY met in New York April 7, 1875. *Pres.* Geo. H. Perine; *Rec. Sec.* N. M. Beckwith;

*American Academy of Dental Science* Meets at Boston, Mass. *Pres.* Dr. D. M. Parker. *Sec.* W. L. Tucker, of Boston ; *Cor. Sec.*, Geo. T. Moffatt.

*Baltimore Dental Society—Pres.* C. T. Brockett; *Rec. Sec.* F. F. Drew.

*Brooklyn Dental Society* Meets semi-monthly. *Pres.* H. G. Mesick. *Rec. Sec* C. P. Crandell ; *Cor. Sec.* Wm. Fishbough.

*Buffalo Dental Society*Meets second Monday of each month at Buffalo. *Pres.* S. A. FREEMAN ; *Rec. Sec.* G. C. DABOLL.

*Boston Dental Institute*—Meets first Tuesday of each month. *Pres.* D. G. WILLIAMS, Buffalo; *Cor. Sec.* D. S. Dickerman.

*California State Dental Association* Meets annually in June. *Pres.* C. C. Knowles, San Francisco; *Rec. Sec.* H. J. Plomteaux, Woodland.

*Chicago Dental Society* Meets monthly at Chicago. *Pres.* C. R. E. Koch; *Rec. Sec.* D. B. Freeman.

*Connecticut Valley Dental Association.* Meets semi-annually, second Tuesday of June and October. *Pres.* H. F. Bishop, Worcester, Mass.; *Rec. Sec.* C. T. Stockwell, Westfield, Mass. Next Meeting in Springfield, Mass., Oct. 17, 1876.

*Connecticut State Dental Society. Pres.* R. C. Dunham, New Britain ; *Rec. Sec.*, D. E. Lane, Hartford.

*Charleston Dental Association*—Meets second Tuesday of every month ; *Pres.* T. F. Chupein ; *Sec. and Treas.* B. A. Muckenfuss.

*Central Penn. Dental Association* Meets at Huntingdon, Jan., 1876. *Pres.* E. J. Green; *Sec.* W. B. Miller.

CINCINNATI DENTAL SOCIETY—Meets first and third Tuesday of each month. *Pres.* A. Berry; *Sec.* A. Downing.

*Delaware Dental Association-* -Meets semi-annually. *Pres.* E. W. Haines, Newark ; *Sec.* C. R. Jeffries, Wilmington.

*Dental Society of the State of Maryland and District of Columbia* Meets on Wednesday, Sept. [illegible], 1876, in Baltimore. *Pres.* R. B. Winder; *Rec. Sec.* R. F. Hunt; *Cor. Sec.* E. P. Keech.

*Eastern Indiana Dental Association.*—Meets semi-annually, first Tuesday in May and last Tuesday in October; *Pres.* J. K. Jameson, Connersville; *Sec.* M. H. Chappee, Knightstown, Ind. (Place of meeting, Connersville.

*Central Ohio Dental Society* Meets at Crestline, on the 2d Monday in April, 1876. *Pres.* M. DeCamp; *Sec.* N. J. Cressinger.

*East Tennessee Dental Association*—Meets at Chattanooga, 3d Wednesday of Sept., 1876. *Pres.* S. M. Prothro; *Rec. Sec.*, S. B. Cooke.

*Georgia State Dental Society* Next meeting at Atlanta, 2d Tuesday in May, 1877. *Pres.* L. D. Carpenter. *Cor. Sec.* Dr. M. S. Jobson.

*Harris Dental Association* Meets quarterly on the first Thursday in February, May. August, Nov'ber. *Pres.* E. K. Young; *Sec.* W. N. Amer, Lancaster.

*Hudson Valley Dental Association* Meets monthly at Troy, N. Y. *Pres.* L. C. Wheeler, *Rec. Sec.* S. G. Andres; *Cor. Sec.* S. D. French.

*Illinois State Dental Soeiety* Meets annually. *Pres.* K. B. Davis, Springfield; *Sec.* E. D. Swain, Chicago. Meets second Tuesday in May, 1877, at Springfield.

*Indiana State Dental Society*—Meets next year at Ft. Wayne, on the last Tuesday of June, 1877. *Pres.* Isaac Knapp, Ft. Wayne; *Sec.* J. E. Cravens.

*Iowa State Dental Society-* -Meets annually. Next meeting at Burlington, on the second Tuesday of May. *Pres.* C. P. Artman Waterloo; *Rec. Sec.* R. L. Cochran Burlington; *Cor. Sec.* I. P. Wilson, Burlington.

*Kansas State Dental Society* Meets annually. Next meeting at Leavanworth, first Wednesday in May, 1877. *Pres.* J. D Patterson, Lawrence; *Sec.* W. N. Shultze, Acheson.

*Kentucky State Dental Association* Meets annually first Tuesday in June, next meeting at Paris. *Pres.* A. O. Rawls, Lexington; *Sec.* A. W. Smith, Richmond.

*Lake Erie Dental Association* Meets Annually. *Pres.* C. B. Ansart, Oil City, Pa. *Sec.* C. D. Elliott, Franklin, Pa. Next meeting first Tuesday of May, 1876.

*Mad River Dental Association* Meets at Xenia on the first Tuesdays in April and October. *Pres.* R. Co[illegible]on, Middletown ; *Sec.* E. Betty, Xenia.

*Maine Dental Society*—Meets in Aug., at Thomaston. *Pres.* E. J. Roberts, Augusta ; *Sec.* C. E. Hussey, Biddeford.

*Maryland Association of Dentists* Meets the last Thursday of every month. *Pres.* J. B. Bean ; *Sec.* S. M. Field.

*Massachusetts Dental Society*—Meets monthly. *Pres.* John T. Codman, Boston; *Rec. Sec.* G. F. Grant, Boston; *Cor. Sec.* T. N, Chandler, Boston.

*Michigan State Dental Association*—Meets annually, next meeting in Detroit in Oct. *Pres.* R. G. Thomas, Detroit; *Rec. & Cor. Sec.* Wm. F. Tremper, Ypsilanti.

*Mississippi Valley Dental Association* Meets annually at Cincinnati, on first Wednesday in March, 1876. *Pres.* H. J. McKellops, St. Louis, Mo.; *Rec. Sec.* Wm. Taft, Cin.
*Mississippi State Dental Association* will meet 3d Wednesday of Aug. '77. *Pres.* J. B. Askew; *Sec.* A. Riser.
*Minnesota Dental Association* Meets annually. Next meeting at Minneapolis; May, 1876. *Pres.* F. A. Williamson, Reading, *Sec.* W. F. Lewis, Winona-St. Paul.
*Missouri Dental Association* Meets on the first Tuesday of June, in St. Louis. *Pres.* C. W. Rivers, St. Louis; *Sec.* W. H. Evans, St. Louis.
*Missouri Valley Dental Association* Meets on the fourth Tuesday in July, 1876, in Omaha, Neb. *Pres.* J. W. Chadduck; *Sec. and Treas.* F. M. Shrirer, Tabor, Iowa.
*Merrimac Valley Dental Association* Meets on the last Thursday of May, 1877, at Portland. *Pres.* A. M. Dudley, Salem, Mass; *Rec. Sec.* W. E. Riggs, Lawrence, Mass., *Cor. Sec.* J. H. Kidder, Lawrence, Mass.
*Memphis Dental Society*—Meets on the first Tuesday of each month. *Pres.* W. M. Arrington; *Sec.* V. F. Elliott; *Treas.* C. L. Harris.

*Nashville Dental Association*—Meets on the second Tuesday of each month. *Pres.* J. C. Ross; *Sec.* R. R. Freeman; *Cor. Sec.* S. J. Cobb.
*New Jersey State Dental Society*—Meets annually. Next meeting at Atlantic City, on the second Tuesday in July, 1876. *Pres.* C. S. Stockton, Newark; *Sec.* C. A. Meeker, Newark.

*New Haven Dental Society*--Meets on the first Wednesday of each month at New Haven. *Pres.* J. T. Metcalf, *Rec and Cor. Sec.* C. L. Smith, of New Haven.
*New Orleans Dental Society* Meets monthly. *Pres.* ———; *Rec. Sec.* A. F. McLane.
*Northern Ohio Dental Association.* Meets annually, second Tuesday of June, 1876, at Cleveland. *Pres.* E. J. Way, Sandusky; *Cor. Sec.* C. Buffett, Clinton.
*Northern Iowa Dental Association*—Meets annually, on the second Tuesday in June, 1873. Next meeting at Waterloo; *Pres.* J. N. Abbott, Lancaster, *Rec. Sec.* A. V Eaton, Anamosa; *Cor. Sec.* A. R. Mason, Waterloo.
*North Carolina Dental Association* Meets annually. Next meeting at Greensboro, 2d Tuesday of Aug., 1876. *Pres.* B. F. Arrington; *Sec.* E. L. Hunter.

*Ohio College Dental Association*—Meets annually Feb. 25. at Cincinnati. *Pres.* N. G. McKellops, St. Louis, Mo.; *Rec. Sec.* H. A. Smith, of Cincinnati.
*Ohio State Dental Association*—Meets annually at Columbus. Next meeting, first Wednesday of December, 1876. *Pres.* C. R. Taft, Cincinnati; *Rec. Sec.* H. L Ambler, Cleveland, O.; *Cor. Sec.* A. F. Emminger, Columbus, O.
*Odontographic Society of Pennsylvania*—Meets on the first Wednesday of each month at 8 o'clock, P. M. in the Philadelphia Dental College. *Pres.* F. M. Dixon; *Cor. Sec.* J. H. McQuillen; *Rec. Sec.* E. L. Hewitt.
*Odontological Society of New York City* Meets the second Tuesday of each month. *Pres.* A. L. Northrup; *Rec. Sec.* Wm. Jarvie, Jr.
*Old Colony Dental Association* Meets quarterly.* *Pres.* C. G. Davis; *Rec. Sec.* L. W. Puffer, North Bridgewater, Mass; *Cor. Sec.* N. C. Fowler, Yarmouth, Mass.
*Ohio Valley Dental Association* Meets annually, in N. E. Ky; this year in Paris, Ky. Tuesday, Sept. 9th.
*Oregon State Dental Society* Meets annually. *Pres.* J. R. Cardwell, Portland; *Rec. Sec.* G. N. Chance, Portland.

*Pennsylvania State Dental Society* meets next in Minnequa Sprins, 2d Tuesday of July, 1877. *Pres.* Robert Hovey; *Rec. Sec.* Jos. Pettit; *Cor. Sec.* W. H. Truman.
*Pennsylvania Association of Dental Surgery* Meets monthly in Philadelphia. *Pres* Spencer Roberts; *Sec.* G. Pettit.
PITTSBURG DENTAL SOCIETY—Meets second Tuesday of each month. *Pres.* W. F Fundenburg; *Sec.* M. L. King.

*San Francisco Dental Association* Meets monthly. *Pres.* C. C. Knowles; *Cor. Se* H. E. Knox; *Rec. Sec.* Wm. Dutch.
*St. Louis Dental Society* Meets monthly. *Pres.* W. H. Morrison; *Sec.* A. H. Fuller.
*Susquehanna Dental Association* Meets annually. *Pres.* W. H. Wissimer, Williamsport, Pa.; *Rec. Sec.* W H. Hertz. Meets May, 1876, at Pittston.
*Society of Dental Surgeons of the City of New York* Meets semi-monthly in New York. *Pres.* B. F. Franklin: *Rec. Sec.* J. S. Latimer; *Cor. Sec.* E. H. Burras.
**Southern Dental Association* Meets annually. Next meeting at Nashville, Tenn, at time of Mardi-Gras. *Pres.* W. G. Redman, La.; *Rec. Sec.* J. F Thompson. Va
*South Carolina State Dental Association* Meets annually. *Pres.* G. W. Norwood, *Sec.* G. F. S. Wright, *Cor. Sec.* N. D. Wilson. Next meeting at Columbia, the first Tuesday of June, 1877.

*Tennessee Dental Association* Meets semi-annually. Next meeting in Nashville, on the 4th Wednesday in June. 1876. *Pres.* R. R. Freeman; *Rec. Sec.* W. H. Morgan *Cor. Sec.* L. G. Noel.

*Virginia State Dental Association* Meets in Richmond, first Thursday in Nov. o each year. *Pres.* H. M. Grant, Abington: *Sec.* G. F. Keesee, Richmond.

*Washington, City Dental Society* Meets annually. *Pres.*, R. F. Hunt, *Sec.* Dr. Evans
*Wisconsin State Dental Association* Meets annually. Next meeting at Milwaukee second Tuesday of July, 1876. *Pres.* R. S. Wells, Sparta; M. T. Moore, La rosse.

### State and District Dental Societies of State of New York.

*New York State Dental Society*--Meets at Albany on the second Wednesday of July next. *Pres.* W. C. Barrett, Warsaw, N. Y.; *Cor. Sec.* S. B. Palmer; *Rec. Sec.* S. S. A. Freeman, Buffalo.

*First District* Meets second and fourth Wednesday evenings of each month. Annual meeting in New York, first Tuesday in June. *Pres.* J. S. Latimer New York, *Rec. Sec.* F. M. Odell, New York.

*Second District.* Annual meeting in Brooklyn, first Monday in April. *Pres.* W. S. Elliott; *Rac. Sec*, M. E. Elmandorf. Brooklyn; *Cor. Sec.* F. W. Dalbare.

*Third District.* Meets semi-annually on second Tuesday in January and July. Annual meeting at Albany on second Tuesday in January. *Pres.* S. P. Welsh; *Rec. Sec.* H. A. Hall, Troy; *Cor. Sec.* J. A. Perkins, Albany.

*Fourth District.* Meets semi-annually. Meets annually at Saratoga on second Tnesday in June. *Pres.* C, A. Elmore, Ft. Edward; *Rec. Sec.* E. S. Pearsall, Saratoga.

*Fifth District.* Meets semi-annually. Annual meeting at Syracuse, second Tuesday in June. *Pres.* F. D. Nellis, Syracuse; *Rec. Sec.* J. S. Marshall, Syracuse; *Cor. Sec.* A. B. Cowles, Rome.

*Sixth District.* Meets semi-annually. Annual meeting at Binghampton on second Tuesday in June, *Pres.* Frank B. Darby, Oswego; *Rec. Sec.* F. E. Perry, Norwich *Cor. Sec.* A. M. Holmes, Morrisville.

*Seventh District*, Meets semi-annually. Annual meeting at Rochester first Tuesday In June. *Pres.* H. S. Miller; *Rec. Sec.* J. E. Line, Rochester.

*Eighth District.* Meets semi-annually. Annual on second Tuesday in May. *Pres.* G. C. Daboll, *Rec. Sec.* S. A. Freeman, *Cor. Sec.* T. G. Lewis, Buffalo.

*The Seventh and Eighth District Societies* hold a union meeting on the last Tueday of October, alternately at Buffalo and Rochester.

The Ohio State Board of Dental Examiners will meet in Cincinnati on the first Wednesday of March 1876, at 12 o'clock. *Pres.* J. Taft; *Sec.* W. P. Horton.

### Exchanges.

ST. LOUIS MEDICAL AND SURGICAL JOURNAL.
BOSTON MEDICAL AND SURGICAL JOURNAL.
THE PACIFIC MEDICAL AND SURGICAL JOURNAL, SAN FRANCISCO.
PHILADELPHIA MEDICAL AND SURGICAL JOURNAL.
CINCINNATI MEDICAL ADVANCE.
CINCINNATI LANCET AND OBSERVER.
DENTAL COSMOS, PHILADELPHIA.
LADIES' REPOSITORY, CINCINNATI.
HALL'S JOURNAL OF HEALTH, N. Y.
CHICAGO MEDICAL EXAMINER.
THE DETROIT REVIEW OF MEDICINE AND PHARMACY.
MEDICAL RECORD, N. Y.
NASHVILLE JOURNAL OF MEDICINE AND SURGERY.
AMERICAN JOURNAL OF DENTAL SCIENCE, BALTIMORE.
THE UNITED STATES MEDICAL AND SURGICAL JOURNAL, CHICAGO.
NEW ORLEANS JOURNAL OF MEDICINE.
AMERICAN AGRICULTURIST, N. Y.
BOSTON JOURNAL OF CHEMISTRY.
JOURNAL OF APPLIED CHEMISTRY, N. Y.
CANADA JOURNAL OF DENTAL SCIENCE, MONTREAL.
INDIANA JOURNAL OF MEDICINE.
MEDICAL AND SURGICAL REPORTER, SALEM, OREGON.
MISSOURI DENTAL JOURNAL, ST. LOUIS.
ECLECTIC MEDICAL JOURNAL, CINCINNATI.

### HARVARD UNIVERSITY.

### Dental Department.

BOSTON, MASS. 1875–76.

### FACULTY.

Charles William Eliot, L.L.D., *President of the University.*
Oliver W. Holmes, M.D., Professor of Anatomy.
Henry J. Bigelow, M.D., Professor of Surgery and Clinical Surgery.
Thomas H. Chandler, D.M.D., Professor of Mechanical Dentistry.
George T. Moffatt, M.D., D.M.D., Professor of Operative Dentistry.
Luther D. Shepard, D.D.S., Adjunct Professor of Operative Dentistry.
Nathaniel W. Hawes, Assistant Professor of Operative Dentistry.
Edward S. Wood, M.D., Assistant Professor of Chemistry.
Henry P. Bowditch, M.D., Assistant Professor of Physiology.
William H. Rollins, D.M.D., Instructor in Dental Pathology.
Charles A. Bracket, D.M.D., Instructor in Dental Therapeutics.
Ira A. Salmon, D.D.S., University Lecturer on Operative Dentistry.
Charles B. Porter, M.D., Demonstrator of Practical Anatomy.
Charles Wilson, D.M.D., Demonstrator in Charge.
George F. Grant, D.M.D., Demonstrator of Mechanical Dentistry.

The plan of study of this School has been radically changed. Instead of the usual oral examinations held at the end of the course, written examinations are had at the end of each year on the subjects pursued during the year, and instead of the customary repetition each year of the lectures and studies of the preceding year, the course is progressive over a period of two years, and the student is required to pass a satisfactory examination in the first year studies before being allowed to proceed to the next. At the end of the course a satisfactory examination must be passed in all the subjects.

The new year begins Sept. 27th, 1877, and ends the last Wednesday in June, 1878. It is divided into two equal terms, with a recess of one week at Christmas.

The regular examinations will be held in the following order, viz.: At the end of the first year, anatomy, including dissection, physiology and general chemistry. A certificate from the demonstrator of anatomy will be required of each student, that he has satisfactorily dissected the three parts of the body.

At the end of the second year, dental pathology, including a knowledge of gestation and diseases of women so far as they affect the mouth and throat, dental materia medica and therapeutics, oral surgery and surgical pathology, operative and mechanical dentistry. The examinations in operative and mechanical dentistry will include actual operations and the preparation of specimens of mechanical dentistry.

Every candidate must be twenty-one years of age, and of good moral character; he must give evidence of having studied medicine or dentistry three full years; he must have spent at least one continuous year at this school, have presented a satisfactory thesis and have passed all the required examinations.

The University Degree, D.M.D. (*Dentariæ Medicinæ Doctor*), is conferred upon those who fulfil the requirements.

Students may be admitted to advanced standing upon passing a satisfactory examination in a majority of the studies already pursued by the class; but no student shall advance with his class or be admitted to advanced standing until he has passed such examination.

The faculty recommend young men who propose to take the degree to spend the whole of the required term of three years of study in the school. But those who wish to spend but two of the three years in the school are earnestly advised to pass their first year of study, before entering, under the direction of a *competent* private instructor.

Unsurpassed facilities for operative and mechanical practice are furnished by the infirmary of the Massachusetts General Hospital which is open throughout the year.

### FEES.

There are no fees for matriculation, for the diploma, nor for the demonstrators. For the first year a student is a member of the school, the fee is $200; for the second year, $150; for any subsequent year, $50.

For further information address,

**THOS. H. CHANDLER, Dean,**

**222 Tremont Street, Boston, Mass.**

tf

### The Dental College
OF THE
### UNIVERSITY of MICHIGAN

The Second Annual Session of this Institution will commence on the 1st of October and close on the last Wednesday of March, thus making a course of six months. The regular course of instruction commences at once and will proceed through the term with the customary holiday vacation.

The students in this department will receive instructions in Anatomy, Physiology, Pathology, Chemistry, Materia Medica, Therapeutics and Surgery, from the professors of their respective branches in the *Department of Medicine* and Surgery of the University, when lectures commence and continue the same as with the Dental College.

### FACULTY OF THE DENTAL DEPARTMENT.

J. B. Angell, LL. D., President.

J. Taft, D. D. S., Professor of Principles aud Practice of Operative Dentistry.

J. A. Watling, D. D, S., Professor of Clinical and Mechanical Dentistry.

W. H. Jackson, Demonstrator of Mechanical Dentistry.

It is desirable that students should be present at the opening of the session, as the regular course of instruction begins at that time. Seats in the lecture-room are assigned by selection to students in the order of registration on the steward's books; and each student is expected to occupy during the session such seat as he may select. Students on arriving in Ann Arbor should call at the steward's office.

### CONDITION OF GRADUATION.

The candidate must be twenty-one years of age. He must furnish evidence of good moral character.

He must devote three years in the study of his profession, in connection with attendance upon a course of medical lectures. He must attend two full courses of lectures in the Dental College, or one course elsewhere and the last one here, and we recommend that he attend three courses regularly.

He must sustain an examination satisfactory to the Faculty in all the branches taught.

A graduate of the Medical College may enter the senior class, and, if found qualified, may graduate after one year has been devoted to the study of dentistry.

Fees and Expenses.—The fees which must be paid in advance, are as follows:

Matriculation Fee.—Residents of Michigan, $10.00; non-residents, $15.00.

Annual Dues.—Residents of Michigan, $15.00; non-residents, $20.00.

Graduation Fee.—For all alike $5.00. The admission fee is paid, but once, and entitles the students to the privileges of permanent membership in any department of the University. The annual tax is paid the first year and every year after.

For further particulars address the Dean of the Dental College, Ann Arbor, Michigan.

tf Feb-4t J. TAFT, Dean.

### HARVARD UNIVERSITY.

### Dental Department.

BOSTON, MASS. 1876-77.

### FACULTY.

Charles William Eliot, L.L.D., *President of the University.*
Oliver W. Holmes, M.D., Professor of Anatomy.
Henry J. Bigelow, M.D., Professor of Surgery and Clinical Surgery.
Thomas H. Chandler, D.M.D., Professor of Mechanical Dentistry.
George T. Moffatt, M.D., D.M.D., Professor of Operative Dentistry.
Luther D. Shepard, D.D.S., Adjunct Professor of Operative Dentistry.
Nathaniel W. Hawes, Assistant Professor of Operative Dentistry.
Edward S. Wood, M.D., Assistant Professor of Chemistry.
Henry P. Bowditch, M.D., Assistant Professor of Physiology.
William H. Rollins, D.M.D , Instructor in Dental Pathology.
Charles A. Bracket, D.M.D., Instructor in Dental Therapeutics.
Ira A. Salmon, D.D.S., University Lecturer on Operative Dentistry.
Charles B. Porter, M.D., Demonstrator of Practical Anatomy.
Charles Wilson, D.M.D., Demonstrator in Charge.
George F. Grant, D.M.D., Demonstrator of Mechanical Dentistry.

The plan of study of this School has been radically changed. Instead of the usual oral examinations held at the end of the course, written examinations are had at the end of each year on the subjects pursued during the year, and instead of the customary repetition each year of the lectures and studies of the preceding year, the course is progressive over a period of two years, and the student is required to pass a satisfactory examination in the first year studies before being allowed to proceed to the next. At the end of the course a satisfactory examination must be passed in all the subjects.

The new year begins Sept. 27th, 1877, and ends the last Wednesday in June, 1878. It is divided into two equal terms, with a recess of one week at Christmas.

The regular examinations will be held in the following order, viz.: At the end of the first year, anatomy, including dissection, physiology and general chemistry. A certificate from the demonstrator of anatomy will be required of each student, that he has satisfactorily dissected the three parts of the body.

At the end of the second year, dental pathology, including a knowledge of gestation and diseases of women so far as they affect the mouth and throat, dental materia medica and therapeutics, oral surgery and surgical pathology, operative and mechanical dentistry. The examinations in operative and mechanical dentistry will include actual operations and the preparation of specimens of mechanical dentistry.

Every candidate must be twenty-one years of age, and of good moral character; he must give evidence of having studied medicine or dentistry three full years; he must have spent at least one continuous year at this school, have presented a satisfactory thesis and have passed all the required examinations.

The University Degree, D.M.D. (*Dentariæ Medicinæ Doctor*), is conferred upon those who fulfil the requirements.

Students may be admitted to advanced standing upon passing a satisfactory examination in a majority of the studies already pursued by the class; but no student shall advance with his class or be admitted to advanced standing until he has passed such examination.

The faculty recommend young men who propose to take the degree to spend the whole of the required term of three years of study in the school. But those who wish to spend but two of the three years in the school are earnestly advised to pass their first year of study, before entering, under the direction of a *competent* private instructor.

Unsurpassed facilities for operative and mechanical practice are furnished by the infirmary of the Massachusetts General Hospital which is open throughout the year.

### FEES.

There are no fees for matriculation, for the diploma, nor for the demonstrators. For the first year a student is a member of the school, the fee is $200; for the second year, $150; for any subsequent year, $50.

For further information address,

**THOS. H. CHANDLER, Dean,**
**222 Tremont Street, Boston, Mass.**

Mar-5

### The Dental College
OF THE
### UNIVERSITY of MICHIGAN

The Second Annual Session of this Institution will commence on the 1st of October and close on the last Wednesday of March, thus making a course of six months. The regular course of instruction commences at once and will proceed through the term with the customary holiday vacation.

The students in this department will receive instructions in Anatomy, Physiology, Pathology, Chemistry, Materia Medica, Therapeutics and Surgery, from the professors of their respective branches in the *Department of Medicine* and Surgery of the University, when lectures commence and continue the same as with the Dental College.

### FACULTY OF THE DENTAL DEPARTMENT.

J. B. Angell, LL. D., President.

J. Taft, D. D. S., Professor of Principles aud Practice of Operative Dentistry.

J. A. Watling, D. D, S., Professor of Clinical and Mechanical Dentistry.

W. H. Jackson, Demonstrator of Mechanical Dentistry.

It is desirable that students should be present at the opening of the session, as the regular course of instruction begins at that time. Seats in the lecture-room are assigned by selection to students in the order of registration on the steward's books; and each student is expected to occupy during the session such seat as he may select. Students on arriving in Ann Arbor should call at the steward's office.

### CONDITION OF GRADUATION.

The candidate must be twenty-one years of age. He must furnish evidence of good moral character.

He must devote three years in the study of his profession, in connection with attendance upon a course of medical lectures. He must attend two full courses of lectures in the Dental College, or one course elsewhere and the last one here, and we recommend that he attend three courses regularly.

He must sustain an examination satisfactory to the Faculty in all the branches taught.

A graduate of the Medical College may enter the senior class, and, if found qualified, may graduate after one year has been devoted to the study of dentistry.

Fees and Expenses.—The fees which must be paid in advance, are as follows:

Matriculation Fee.—Residents of Michigan, $10.00; non-residents, $15.00.

Annual Dues.—Residents of Michigan, $15.00; non-residents, $20.00.

Graduation Fee.—For all alike $5.00. The admission fee is paid, but once, and entitles the students to the privileges of permanent membership in any department of the University. The annual tax is paid the first year and every year after.

For further particulars address the Dean of the Dental College, Ann Arbor, Michigan.

tf J. TAFT, Dean.

### HARVARD UNIVERSITY.

### Dental Department.

### BOSTON, MASS. 1877-78.

---

### FACULTY.

CHARLES WILLIAM ELIOT, L.L.D., *President of the University.*
OLIVER W. HOLMES, M.D., Professor of Anatomy.
HENRY J. BIGELOW, M.D., Professor of Surgery and Clinical Surgery.
THOMAS H. CHANDLER, D.M.D., Professor of Mechanical Dentistry.
GEORGE T. MOFFATT, M.D., D.M.D., Professor of Operative Dentistry.
LUTHER D. SHEPARD, D.D.S., Adjunct Professor of Operative Dentistry.
NATHANIEL W. HAWES, Assistant Professor of Operative Dentistry.
EDWARD S. WOOD, M.D., Assistant Professor of Chemistry.
HENRY P. BOWDITCH, M.D., Assistant Professor of Physiology.
WILLIAM H. ROLLINS, D.M.D, Instructor in Dental Pathology.
CHARLES A. BRACKET, D.M.D., Instructor in Dental Therapeutics.
IRA A. SALMON, D.D.S., University Lecturer on Operative Dentistry.
CHARLES B. PORTER, M.D., Demonstrator of Practical Anatomy.
CHARLES WILSON, D.M.D., Demonstrator in Charge.
GEORGE F. GRANT, D.M.D., Demonstrator of Mechanical Dentistry.

---

The plan of study of this School has been radically changed. Instead of the usual oral examinations held at the end of the course, written examinations are had at the end of each year on the subjects pursued during the year, and instead of the customary repetition each year of the lectures and studies of the preceding year, the course is progressive over a period of two years, and the student is required to pass a satisfactory examination in the first year studies before being allowed to proceed to the next. At the end of the course a satisfactory examination must be passed in all the subjects.

The new year begins Sept. 27th, 1877, and ends the last Wednesday in June, 1878. It is divided into two equal terms, with a recess of one week at Christmas.

The regular examinations will be held in the following order, viz.: At the end of the first year, anatomy, including dissection, physiology and general chemistry. A certificate from the demonstrator of anatomy will be required of each student, that he has satisfactorily dissected the three parts of the body.

At the end of the second year, dental pathology, including a knowledge of gestation and diseases of women so far as they affect the mouth and throat, dental materia medica and therapeutics, oral surgery and surgical pathology, operative and mechanical dentistry. The examinations in operative and mechanical dentistry will include actual operations and the preparation of specimens of mechanical dentistry.

Every candidate must be twenty-one years of age, and of good moral character; he must give evidence of having studied medicine or dentistry three full years; he must have spent at least one continuous year at this school, have presented a satisfactory thesis and have passed all the required examinations.

The University Degree, D.M.D. (*Dentariæ Medicinæ Doctor*), is conferred upon those who fulfil the requirements.

Students may be admitted to advanced standing upon passing a satisfactory examination in a majority of the studies already pursued by the class; but no student shall advance with his class or be admitted to advanced standing until he has passed such examination.

The faculty recommend young men who propose to take the degree to spend the whole of the required term of three years of study in the school. But those who wish to spend but two of the three years in the school are earnestly advised to pass their first year of study, before entering, under the direction of a *competent* private instructor.

Unsurpassed facilities for operative and mechanical practice are furnished by the infirmary of the Massachusetts General Hospital which is open throughout the year.

---

### FEES.

There are no fees for matriculation, for the diploma, nor for the demonstrators. For the first year a student is a member of the school, the fee is $200; for the second year, $150; for any subsequent year, $50.

For further information address,

**THOS. H. CHANDLER, Dean,**

**222 Tremont Street, Boston, Mass.**

*American Dental Association*—Meets on the first Tuesday in Aug., 1877, at Chicago; *Pres.* G. W. Keeley, Oxford, Ohio; *Cor. Sec.* J. H. McQuilien, Philadelphia, *Rec. Sec.* C. Stoddard Smith, Springfield, Ill.

*American Dental Convention*—Meets at Deer Park, Md., second Tuesday in August, 1877. *Pres.* W. H. Atkinson, New York; *Cor. Sec.* G. W. Mills, Baltimore; *Rec. Sec.* A. Tees, Philadelphia, Pa.

List of Societies Represented in the American Dental Association:

*American Academy of Dental Surgery*—Meets in New York 187 . *Pres.* Geo. H. Perine; *Rec. Sec.* N. M. Beckwith.

*American Academy of Dental Science*—Meets at Boston, Mass. *Pres.* Dr. D. M. Parker, *Sec.* W. L. Tucker, of Boston: *Cor. Sec.* Geo. T. Moffatt.

*Baltimore Dental Society*—*Pres.* C. T. Brockett; *Rec. Sec.* F. F. Drew.

*Brooklyn Dental Society*—Meets semi-monthly. *Pres.* H. G. Mesick; *Rec. Sec.* C. P. Crandell; *Cor. Sec.* Wm. Fishbough.

*Buffalo Dental Society*—Meets second Monday of each month, at Buffalo. *Pres.* S. A. Freeman; *Rec. Sec.* G. C. Daboll.

*Boston Dental Institute*—Meets first Tuesday of each month. *Pres.* D. G. Williams, Buffalo; *Cor. Sec.* D. S. Dickerman.

*California State Dental Association*—Meets annually in June. *Pres.* Wm. Dutch, San Francisco; *Rec. Sec.* L. L. Dunbar, San Francisco. Next meeting June 5. 1877, in San Francisco.

*Chicago Dental Society*—Meets monthly at Chicago. *Pres.* C. R. E. Koch; *Rec. Sec.* D. B. Freeman.

*Connecticut Valley Dental Association*—Meets semi-annually, second Tuesday of June and October. *Pres.* H. F. Bishop, worcester, Mass.; *Rec. Sec.* C. T. Stockwell, Westfield, Mass. Next meeting in Springfield, Mass., October 17, 1877.

*Connecticut State Dental Society*—*Pres.* R. C. Dunham, New Britain; *Rec. Sec.* D. E. Lane, Hartford.

*Charleston Dental Association*—Meets second Tuesday of every month. *Pres.* T. F. Chupein; *Sec.* and *Treas.* B. A. Muckenfuss.

*Central Pennsylvania Dental Association*—Meets at Huntingdon, Jan., 1878. *Pres.* E. J. Green; *Sec.* W. B. Miller.

*Cincinnati Dental Society*—Meets first and third Tuesday of each month. *Pres.* A. Berry; *Sec.* A. Downing.

*Delaware Dental Association*—Meets semi-annually. *Pres.* E. W. Haines, Newark; *Sec.* C. R. Jeffries, Wilmington

*Dental Society of the State of Maryland and District of Columbia*—Meets on Wednesday, Sept. 6, 1877, in Baltimore. *Pres.* R. B. Winder; *Rec. Sec.* R. F. Hunt; *Cor Sec.* E. P. Keech.

*Eastern Indiana Dental Association*—Meets semi-annually, first Tuesday in May, and last Tuesday in October; *Pres.* J. K. Jameson, Connerville; *Sec.* M. H. Chappee, Knightstown, Ind. Place of meeting. Connersville.

*Central Ohio Dental Society*—Meets at Crestline, on the second Monday in April, 1877. *Pres.* M. DeCamp; *Sec.* N. J. Cressinger.

*East Tennessee Dental Association*—Meets at Chattanooga, third Wednesday of Sept. 1877. *Pres.* S. M. Prothro; *Rec. Sec.* S. B. Cooke.

*Georgia State Dental Society*—Next meeting at Atlanta, second Tuesday in May, 1877. *Pres.* L. D. Carpenter; *Cor. Sec.* Dr. M. S. Jobson.

*Harris Dental Association*—Meets quarterly, on the first Tursday in February, May, August and November. *Pres.* E. K. Young; *Sec.* W. N. amer, Lancaster.

*Hudson Valley Dental Association*—Meets monthly at Troy, N. Y. *Pres.* L. C. Wheeler; *Rec. Sec.* S. G. Andres; *Cor. Sec.* S. D. French.

*Illinois State Dental Society*—Meets annually. *Pres.* K. B. Davis, Springfield; *Sec.* E. D. Swain, chicago. Meets second Tuesday in May, 1877, at Springfield.

*Indiana State Dental Society*—Meets next year as Ft. Wayne, on the last Tuesday of June, 1877. *Pres.* Isaac Knapp, Ft. Wayne; *Sec.* J. E. Cravens.

*Iowa State Dental Society*—Meets annually. Next meeting at Burlington, on the second Tuesday of May. *Pres.* C. P. Artman, Waterloo; *Rec. Sec.* R. L. Cochran, Burlington; *Cor. Sec.* I. P. Wilson, Burlington.

*Kansas State Dental Society*—Meets annually. Next meeting at Leavanworth, first Wednesday in May, 1877. *Pres.* J. D. Patterson, Lawrence; *Sec.* W. N. Shultze, Atchison.

*Kentucky State Dental Association*—Meets annually first Tuesday in June. Next meeting at Paris. *Pres.* A. O. Rawls, Lexington; *Sec.* A. W. Smith, Richmond.

*Lake Erie Dental Association*—Meets annually. *Pres.* C. B. Ansart, Oil City, Penn. *Sec.* C. D. Elliot, Franklin, Penn. Next meeting first Tuesday of May, 1876.

*Mad River Dental Association*—Meets at Xenia on the first Tuesday in April and October. *Pres.* R. Corson, Middletown; *Sec.* E. Betty, Xenia.

*Maine Dental Society*—Meets in August, at Thomaston. *Pres.* E. J. Roberts, Augusta, *Sec.* C. E. Hussey, Biddeford.

*Maryland Association of Dentists*—Meets the last Thursday of every month. *Pres.* J. B. Bean; *Sec.* S. M. Field.

*Massachusetts Dental Society*—Meets monthly. *Pres.* John T. Codman, Boston; *Rec. Sec.* G. F. Grant, Boston; *Cor. Sec.* T. N. Chandler, Boston.

*Michigan State Dental Association*—Meets annually. Next meeting at Ann Arbor,

October 9. *Pres.* R. G. Thomas, Detroit; *Sec.* and *Treas.* E. S. Holmes, Grand Rapids.

*Missippi Valley Dental Association*—Meets annually at Cincinnati, on first Wednesday in March, 1878. *Pres.* A. O. Rawls, Lexington, Ky.; *Rec. Sec.* Wm. Taft, Cincinnati.

*Mississippi State Dental Association*—Will meet third Wednesday of August, 1877. *Pres.* J. B. Askew ; *Sec.* A. Riser.

*Minnesota Dental Association*—Meets annually. Next meeting at Minneapolis, May, 1877. *Pres.* F. A. Williamson, Reading; *Sec.* W. F. Lewis, Winona and St. Paul.

*Missouri Dental Association*—Meets on the first Tuesday in June, in St. Louis. *Pres.* C. W. Rivers, St. Louis; *Sec.* W. H. Evans, St. Louis.

*Missouri Valley Dental Association*—Meets on the fourth Tuesday in July, 1876, in Omaha, Neb. *Pres.* J. W. Chadduck; *Sec.* and *Treas.* F. M. Shrirer, Tabor, Iowa.

*Merrimac Valley Dental Association*—Meets on the 3d and 4th of May, 1877, at Lynn. *Pres.* H. Hill, Manchester, N. H.; *Rec. Sec.* W. E. Riggs, Lawrence, Mass.; *Cor. Sec.* J. H. Kidder, Lawrence, Mass.

*Memphis Dental Society*—Meets on the first Tuesday of each month. *Pres.* W. M. Arrington; *Sec.* V. F. Elliot; *Treas.* C. L. Harris.

*Nashville Dental Association*—Meets on the second Tuesday of each month. *Pres.* J. C. Ross; *Sec.* R. R. Freeman; *Cor. Sec.* S. J. Cobb.

*New Jersey State Dental Society*—Meets annually. Next meeting at Atlantic City, on the second Tuesday in July, 1877. *Pres.* C. S. Stockton, Newark; *Sec.* C. A. Meeker, Newark.

*New Haven Dental Society*—Meets on the first Wednesday of each month at New Haven. *Pres.* J. T. Metcalf; *Rec.* and *Cor. Sec.* C. L. Smith, of New Haven.

*New Orleans Dental Society*—Meets on the first Thursday of each month. *Pres.* W. S. Chandler; *Rec. Sec.* F. J. Knapp.

*Northern Ohio Dental Association*—Meets annually, second Tuesday of June, 1876, at Cleaveland. *Pres.* E. J. Way, Sandusky; *Cor. Sec.* C. Buffett, Clinton.

*Northern Iowa Dental Asssociation*—Meets annually, on the second Tuesday in June, 1873. Next meeting at waterloo. *Pres.* J. N. Abbott Lancaster; *Rec. Sec.* A. V. Eaton. Anamosa; *Cor. Sec.* A. R. Mason, Waterloo.

*North Carolina Dental Association*—Meets annually. Next meeting at Greensboro, second Tuesday of August, 1876. *Pres.* B. F. Arrington; *Sec.* E. L. Hunter.

*Ohio College Dental Association*—Meets annually, February 25, at Cincinnati. *Pres.* N. G. McKellops, St. Louis, Mo.; *Rec. Sec.* H. A. Smith, of Cincinnati.

*Ohio State Dental Association*—Meets annually at Columbus. Next meeting first Wednesday of December, 1876. *Pres.* C. R. Taft, Cincinnati; *Rec. Sec.* H. L. Ambler, Cleveland, Ohio; *Cor. Sec.* A. F. Emminger, Columbus, Ohio.

*Odontographic Society of Pennsylvania*—Meets on the first Wednesday of each month, at 8 o'clock, P.M., in the Philadelphia Dental College. *Pres.* F. M. Dixon; *Cor. Sec.* J. H. McQuillen; *Rec. Sec.* E. L. Hewitt.

*Odontological Society of New York City*—Meets the second Tuesday of each month. *Pres.* A. L. Northrup; *Rec. Sec.* Wm. Jarvie, Jr.

*Old Colony Dental Association*—Meets quarterly.* *Pres.* C. G. Davis; *Rec. Sec.* L. W. Puffer, North Bridgewater, Mass.; *Cor. Sec.* N. C. Fowler, Yarmouth, Mass.

*Ohio Valley Dental Association*—Meets annually in N. E., Ky.; this year in Paris, Ky., Tuesday, September 9th.

*Oregon State Dental Society*—Meets annually. *Pres.* J. R. Cardwell, Portland; *Rec. Sec.* G. N. Chance, Portland.

*Pennsylvania State Dental Society*—Meets next in Minnequa Springs, second Tuesday of July, 1877. *Pres.* Robert Hovey; *Rec. Sec.* Jos. Pettit; *Cor Sec.* W. H. Truman.

*Pennsylvania Association of Dental Surgeons*—Meets monthly in Philadelphia. *Pres.* Robert Henry; *Sec.* G. Pettit.

*Pittsburg Dental Society*—Meets second Tuesday of each month. *Pres.* W. F. Fundenburg; *Sec.* M. L. King.

*San Francisco Dental Association*—Meets monthly. *Pres.* C. C. Knowles; *Cor. Sec.* H. E. Knox; *Rec. Sec.* Wm. Dutch.

*St. Louis Dental Society*—Meets monthly. *Pres.* W. H. Morrison; *Sec.* A. H. Fuller.

*Susquehanna Dental Association*—Meets annually. *Pres.* W. H. Wissimer, Williamsport, Penn.; *Rec. Sec.* W. H. Hertz. Meets May, 1876, at Pittston.

*Society of Dental Surgeons of the City of New York*—Meets semi-monthly in New York. *Pres.* B. F. Franklin; *Rec. Sec.* J. S. Latimer; *Cor. Sec.* E. H. Burras.

**Southern Dental Association*—Meets annually. Next meeting at Nashville, Tenn., at time of Mardi-Gras. *Pres.* W. G. Redman, La.; *Rec. Sec.* J. F. Thompson, Va.

*South Carolina State Dental Association*—Meets annually. *Pres.* G. F. S. Wright, Columbia; *Sec.* T. F. Cheupein, Charleston; *Cor. Sec* J. S. Thompson, Abbeville. Next meeting at Columbia, the first Tuesday of June, 1877.

*Tennessee Dental Association*—Meets semi-annually. Next meeting at Lookout Mountain on the fourth Wednesday in June, 1877. *Pres.* R. R. Freeman; *Rec. Sec.* W. H. Morgan; *Cor. Sec.* L. G. Noel.

*Virginia State Dental Association*—Meets in Richmond first Tusday in November of each year. *Pres.* H. M. Grant, Abington; *Sec.* G. F. Keesee, Richmond.

*Washington City Dental Society*—Meets annually. *Pres.* R. F. Hunt; *Sec.* Dr. Evans.

*Wisconsin State Dental Association*—Meets annually. Next meeting at Milwaukee, second Tuesday of July, 1876. *Pres.* R. S. Wells, Sparta; *Sec.* M. T. Moore, La Crosse.

### State and District Dental Societies of State of New York.

*New York State Dental Society*—Meets at Albany on the second Wednesday of July next *Pres.* W. C. Barrett, Warsaw, N. Y.; *Cor. Sec.* S. B. Palmer; *Rec. Sec.* S. S. A. Freeman, Buffalo.

*First District*—Meets second and fourth Wednesday evenings of each month. Annual meeting in New York, first Tuesday in June. *Pres.* J. S. Latimer, New York; *Rec. Sec.* F. M. Odell, New York.

*Second District*—Annual meeting in Brooklyn, first Monday in April. *Pres.* C. D. Cook; *Rec. Sec.* G. L. Mason, Brooklyn; *Cor. Sec.* W. S. Elliott. The meetings are held quarterly, second Thursday in January, April, July and October.

*Third District*—Meets semi-annually on second Tuesday in January and July. Annual meeting at Albany on second Tuesday in January. *Pres.* S. P. Welsh; *Rec. Sec.* H. A. Hall, Troy; *Cor. Sec.* J. A. Perkins, Albany.

*Fourth District*—Meets semi-annually. Meets annually at Saratoga on second Tuesday in June. *Pres.* C. A. Elmore, Ft. Edward; *Rec. Sec.* E. S. Pearsall, Saratoga.

*Fifth District*—Meets semi-annually. Annual meeting at Syracuse, second Tuesday in June. *Pres.* F. D. Nellis, Syracuse; *Rec. Sec.* J. S. Marshall, Syracuse; *Cor. Sec.* A. B. Cowles, Rome.

*Sixth District*—Meets semi-annually. Aunual meeting at Binghampton, on second Tuesday in June. *Pres.* Frank B. Darby, Oswego; *Rec. Sec.* F. E. Perry, Norwich; *Cor. Sec.* A. M. Holmes, Morrisville.

*Seventh District*—Meets semi-annually. Annual meeting at Rochester, first Tuesday in June. *Pres.* H. S. Miller; *Rec. Sec.* J. E. Line, Rochester.

*Eighth District*—Meets semi-annually. Annual meeting on second Tuesday in May. *Pres.* G. C. Daboll; *Rec. Sec.* S. A. Freeman; *Cor. Sec.* T. G. Lewis, Buffalo.

*The Seventh and Eighth District Societies*—Hold a union meeting on the last Tuesday of October, alternately at Buffalo and Rochester.

*The Ohio State Board of Dental Examiners*—Will meet in Cincinnati on the first Wednesday of March, 1876, at 12 o'clock. *Pres.* J. Taft; *Sec.* W. P. Horton.

### EXCHANGES.

ST. LOUIS MEDICAL AND SURGICAL JOURNAL.
BOSTON MEDICAL AND SURGICAL JOURNAL.
THE PACIFIC MEDICAL AND SURGICAL JOURNAL, SAN FRANCISCO.
PHILADELPHIA MEDICAL AND SULGICAL JNURNAL.
CINCINNATI MEDICAL ADVANCE.
CINCINNATI LANCET AND OBSERVER.
DENTAL COSMOS, PHILADELPHIA.
LADIES' REPOSITORY, CINCINNATI.
CHICAGO MEDICAL EXAMINER.
THE DETROIT REVIEW OF MEDICINE AND PHARMACY.
MEDICAL RECORD, N. Y.
NASHVILLE JOURNAL OF MEDICINE AND SURGERY.
AMERICAN JOURNAL OF DENTAL SCIENCE, BALTIMORE.
THE UNITED STATES MEDICAL AND SURGICAL JOURNAL, CHICAGO.
BOSTON JOURNAL OF CHEMISTRY.
JOURNAL OF APPLIED CHEMISTRY.
CANADA JOURNAL OF DENTAL SCIENCE, MONTREAL.
INDIANA JOURNAL OF MEDICINE.
MEDICAL AND SURGICAL REPORTER, SALEM, OREGON.
MISSOURI DENTAL JOURNAL. ST. LOUIS.
ECLECTIC MEDICAL JOURNAL, CINCINNATI.

### HARVARD UNIVERSITY.
### Dental Department.
### BOSTON, MASS. 1877-78.

---

**FACULTY.**

Charles William Eliot, L.L.D., *President of the University.*
Oliver W. Holmes, M.D., Professor of Anatomy.
Henry J. Bigelow, M.D., Professor of Surgery and Clinical Surgery.
Thomas H. Chandler, D.M.D., Professor of Mechanical Dentistry.
George T. Moffatt, M.D., D.M.D., Professor of Operative Dentistry.
Luther D. Shepard, D.D.S., Adjunct Professor of Operative Dentistry.
Nathaniel W. Hawes, Assistant Professor of Operative Dentistry.
Edward S. Wood, M.D., Assistant Professor of Chemistry.
Henry P. Bowditch, M.D., Assistant Professor of Physiology.
William H. Rollins, D.M.D, Instructor in Dental Pathology.
Charles A. Bracket, D.M.D., Instructor in Dental Therapeutics.
Ira A. Salmon, D.D.S., University Lecturer on Operative Dentistry.
Charles B. Porter, M.D., Demonstrator of Practical Anatomy.
Charles Wilson, D.M.D., Demonstrator in Charge.
George F. Grant, D.M.D., Demonstrator of Mechanical Dentistry.

---

The plan of study of this School has been radically changed. Instead of the usual oral examinations held at the end of the course, written examinations are had at the end of each year on the subjects pursued during the year, and instead of the customary repetition each year of the lectures and studies of the preceding year, the course is progressive over a period of two years, and the student is required to pass a satisfactory examination in the first year studies before being allowed to proceed to the next. At the end of the course a satisfactory examination must be passed in all the subjects.

The new year begins Sept. 27th, 1877, and ends the last Wednesday in June, 1878. It is divided into two equal terms, with a recess of one week at Christmas.

The regular examinations will be held in the following order, viz.: At the end of the first year, anatomy, including dissection, physiology and general chemistry. A certificate from the demonstrator of anatomy will be required of each student, that he has satisfactorily dissected the three parts of the body.

At the end of the second year, dental pathology, including a knowledge of gestation and diseases of women so far as they affect the mouth and throat, dental materia medica and therapeutics, oral surgery and surgical pathology, operative and mechanical dentistry. The examinations in operative and mechanical dentistry will include actual operations and the preparation of specimens of mechanical dentistry.

Every candidate must be twenty-one years of age, and of good moral character; he must give evidence of having studied medicine or dentistry three full years; he must have spent at least one continuous year at this school, have presented a satisfactory thesis and have passed all the required examinations.

The University Degree, D.M.D. (*Dentariæ Medicinæ Doctor*), is conferred upon those who fulfil the requirements.

Students may be admitted to advanced standing upon passing a satisfactory examination in a majority of the studies already pursued by the class; but no student shall advance with his class or be admitted to advanced standing until he has passed such examination.

The faculty recommend young men who propose to take the degree to spend the whole of the required term of three years of study in the school. But those who wish to spend but two of the three years in the school are earnestly advised to pass their first year of study, before entering, under the direction of a *competent* private instructor.

Unsurpassed facilities for operative and mechanical practice are furnished by the infirmary of the Massachusetts General Hospital which is open throughout the year.

---

**FEES.**

There are no fees for matriculation, for the diploma, nor for the demonstrators. For the first year a student is a member of the school, the fee is $200; for the second year, $150; for any subsequent year, $50.

For further information address,

**THOS. H. CHANDLER, Dean,**

**222 Tremont Street, Boston, Mass.**

*American Dental Association*—Meets on the first Tuesday in Aug., 1877, at Chicago, *Pres.* G. W. Keeley, Oxford, Ohio; *Cor. Sec.* J. H. McQuilien, Philadelphia; *Rec. Sec.* C. Stoddard Smith, Springfield, Ill.

*American Dental Convention*—Meets at Deer Park, Md., second Tuesday in August, 1877. *Pres.* W. H. Atkinson, New York; *Cor. Sec.* G. W. Mills, Baltimore; *Rec. Sec.* A. Tees, Philadelphia, Pa.

List of Societies Represented in the American Dental Association:

*American Academy of Dental Surgery*—Meets in New York 187 . *Pres.* Geo. H. Perine; *Rec. Sec.* N. M. Beckwith.

*American Academy of Dental Science*—Meets at Boston, Mass. *Pres.* Dr. D. M. Parker, *Sec.* W. L. Tucker, of Boston: *Cor. Sec.* Geo. T. Moffatt.

*Baltimore Dental Society*—*Pres.* C. T. Brockett; *Rec. Sec.* F. F. Drew.

*Brooklyn Dental Society*—Meets semi-monthly. *Pres.* H. G. Merick; *Rec. Sec.* C. P. Crandell.

*Buffalo Dental Society*—Meets second Monday of each month, at Buffalo. *Pres.* S. A. Freeman; *Rec. Sec.* G. C. Daboll.

*Boston Dental Institute*—Meets first Tuesday of each month. *Pres.* D. G. Williams, Buffalo; *Cor. Sec.* D. S. Dickerman.

*California State Dental Association*—Meets annually in June. *Pres.* Wm. Dutch, San Francisco; *Rec. Sec.* L. L. Dunbar, San Francisco. Next meeting June 5. 1877, in San Francisco.

*Chicago Dental Society*—Meets monthly at Chicago. *Pres.* C. R. E. Koch; *Rec. Sec.* D. B. Freeman.

*Connecticut Valley Dental Association*—Meets semi-annually, second Tuesday of June and October. *Pres.* H. F. Bishop, worcester, Mass.; *Rec. Sec.* C. T. Stockwell, Westfield, Mass. Next meeting in Springfield, Mass., October 17, 1877.

*Connecticut State Dental Society*—*Pres.* E. T. Payne; *Rec. Sec.* Geo. L. Parmelee. Hartford. Semi-annual meeting in October in Hartford.

*Charleston Dental Association*—Meets second Tuesday of every month. *Pres.* T. F. Chupein; *Sec.* and *Treas.* B. A. Muckenfuss.

*Central Pennsylvania Dental Association*—Meets at Huntingdon, Jan., 1878. *Pres.* E. J. Green; *Sec.* W. B. Miller.

*Cincinnati Dental Society*—Meets first and third Tuesday of each month. *Pres.* A. Berry; *Sec.* A. Downing.

*Dental Society of the State of Maryland and District of Columbia*—Meets annually. Next meeting at Deer Park, on the second Tuesday in August, 1877. *Pres.* R. F. Hunt, of Washington, D. C.; *Sec.* H. C. Thompson.

*Eastern Indiana Dental Association*—Meets semi-annually, first Tuesday in May, and last Tuesday in October; *Pres.* J. W. Ellis, New Castle; *Sec.* M. H. Chappell, Knightstown, Ind. Place of meeting, Cambridge City.

*Central Ohio Dental Society*—Meets at Crestline, on the second Monday in April, 1877. *Pres.* M. DeCamp; *Sec.* N. J. Cressinger.

*East Tennessee Dental Association*—Meets at Chattanooga, third Wednesday of Sept. 1877. *Pres.* S. M. Prothro; *Rec. Sec.* S. B. Cooke.

*Georgia State Dental Society*—Next meeting at Atlanta, second Tuesday in May, 1877. *Pres.* L. D. Carpenter; *Cor. Sec.* Dr. M. S. Jobson.

*Harris Dental Association*—Meets quarterly. Next Meeting at Columbia, Pa., first Thursday in August. *Pres.* J. D. Heiges; *Sec.* W. N. Amer, Lancaster, Pa.

*Hudson Valley Dental Association*—Meets monthly at Troy, N. Y. *Pres.* L. C. Wheeler; *Rec. Sec.* S. G. Andres; *Cor. Sec.* S. D. French.

*Illinois State Dental Society*—Meets annually. *Pres.* C. R. E. Koch; *Sec.* E. D. Swain, Chicago. Next place of meeting, Rockford, second Tuesday in May, 1878.

*Indiana State Dental Society*—Meets next year as Ft. Wayne, on the last Tuesday of June, 1877. *Pres.* Isaac Knapp, Ft. Wayne; *Sec.* J. E. Cravens.

*Iowa State Dental Society*—Meets annually. Next meeting at Iowa City on the second Tuesday of May. *Pres.* G. S. Shattuck, Belle Plane; *Rec. Sec.* E. E. Hughes of Newton; *Cor. Sec.* I. P. Wilson, Burlington.

*Kansas State Dental Society*—Meets annually. Next meeting at Leavanworth, first Wednesday in May, 1877. *Pres.* J. D. Patterson, Lawrence; *Sec.* W. N. Shultze, Atchison.

*Kentucky State Dental Association*—Meets annually first Tuesday in June. Next meeting at Paris. *Pres.* A. O. Rawls, Lexington; *Sec.* A. W. Smith, Richmond.

*Lake Erie Dental Association*—Meets annually. *Pres.* C. B. Ansart, Oil City, Penn. *Sec.* C. D. Elliot, Franklin, Penn. Next meeting first Tuesday of May, 1876.

*Mad River Dental Association*—Meets at Xenia on the first Tuesday in April and October. *Pres.* R. Corson, Middletown; *Sec.* E. Betty, Xenia.

*Maine Dental Society*—Meets in August, at Thomaston. *Pres.* E. J. Roberts, Augusta, *Sec.* C. E. Hussey, Biddeford.

*Massachusetts Dental Society*—Meets monthly. *Pres.* John T. Codman, Boston; *Rec. Sec.* G. F. Grant, Boston; *Cor. Sec.* T. N. Chandler, Boston.

*Michigan State Dental Association*—Meets annually. Next meeting at Ann Arbor,

October 9. *Pres.* R. G. Thomas, Detroit; *Sec.* and *Treas.* E. S. Holmes, Grand Rapids.

*Missippi Valley Dental Association*—Meets annually at Cincinnati, on first Wednesday in March, 1878. *Pres.* A. O. Rawls, Lexington, Ky.; *Rec. Sec.* Wm. Taft, Cincinnati.

*Mississippi State Dental Association*—Will meet third Wednesday of August, 1877, at Canton. *Pres.* J. B. Askew; *Sec.* A. Riser, Port Gibson.

*Minnesota Dental Association*—Meets annually. Meets May 26, 1878 at Winona. *Pres.* W. F. Lewis; *Sec.* C. M. Bailey, Minneapolis.

*Missouri Dental Association*—Meets on the first Tuesday in June, in St. Louis. *Pres.* C. W. Rivers, St. Louis; *Sec.* W. H. Evans, St. Louis.

*Missouri Valley Dental Association*—Meets on the fourth Tuesday in July, 1876, in Omaha, Neb. *Pres.* J. W. Chadduck; *Sec.* and *Treas.* F. M. Shrirer, Tabor, Iowa.

*Merrimac Valley Dental Association*—Meets on the 3d and 4th of May, 1877, at Lynn. *Pres.* H. Hill, Manchester, N. H.; *Rec. Sec.* W. E. Riggs, Lawrence, Mass.; *Cor. Sec.* J. H. Kidder, Lawrence, Mass.

*Memphis Dental Society*—Meets on the first Tuesday of each month. *Pres.* W. M. Arrington; *Sec.* V. F. Elliot; *Treas.* C. L. Harris.

*Nashville Dental Association*—Meets on the second Tuesday of each month. *Pres.* R. R. Freeman; *Sec.* L. G. Noel, Nashville.

*New Jersey State Dental Society*—Meets annually. Next meeting at Long Branch, on the second Tuesday in July, 1877. *Pres.* T. B. Welch, Newark; *Sec.* C. A. Meeker, Newark.

*New Haven Dental Society*—Meets on the first Wednesday of each month at New Haven. *Pres.* J. T. Metcalf; *Rec.* and *Cor. Sec.* C. L. Smith, of New Haven.

*New Orleans Dental Society*—Meets on the first Thursday of each month. *Pres.* W. S. Chandler; *Rec. Sec.* F. J. Knapp.

*Northern Ohio Dental Association*—Meets annually, second Tuesday of June, 1876, at Cleaveland. *Pres.* E. J. Way, Sandusky; *Cor. Sec.* C. Buffett, Clinton.

*Northern Iowa Dental Association*—Meets annually, on the second Tuesday in June, 1873. Next meeting at waterloo. *Pres.* J. N. Abbott Lancaster; *Rec. Sec.* A. V. Eaton. Anamosa; *Cor. Sec.* A. R. Mason, Waterloo.

*North Carolina Dental Association*—Meets annually. Next meeting at Greensboro, second Tuesday of August, 1876. *Pres.* B. F. Arrington; *Sec.* E. L. Hunter.

*Ohio College Dental Association*—Meets annually, February 25, at Cincinnati. *Pres.* N. G. McKellops, St. Louis, Mo.; *Rec. Sec.* H. A. Smith, of Cincinnati.

*Ohio State Dental Association*—Meets annually at Columbus, first Wednesday of December, 1877. *Pres.* I. Williams; *Rec. Sec.* H. L. Ambler.

*Odontographic Society of Pennsylvania*—Meets on the first Wednesday of each month, at 8 o'clock, P.M., in the Philadelphia Dental College. *Pres.* F. M. Dixon; *Cor. Sec.* J. H. McQuillen; *Rec. Sec.* E. L. Hewitt.

*Odontological Society of New York City*—Meets the second Tuesday of each month. *Pres.* A. L. Northrup; *Rec. Sec.* Wm. Jarvie, Jr.

*Old Colony Dental Association*—Meets quarterly.* *Pres.* C. G. Davis; *Rec. Sec.* L. W. Puffer, North Bridgewater, Mass.; *Cor. Sec.* N. C. Fowler, Yarmouth, Mass.

*Ohio Valley Dental Association*—Meets annually in N. E., Ky.; this year in Paris, Ky., Tuesday, September 9th.

*Oregon State Dental Society*—Meets annually. *Pres.* J. R. Cardwell, Portland; *Rec. Sec.* G. N. Chance, Portland.

*Pennsylvania State Dental Society*—Meets next in Minnequa Springs, second Tuesday of July, 1877. *Pres.* Robert Hovey; *Rec. Sec.* Jos. Pettit; *Cor Sec.* W. H. Truman.

*Pennsylvania Association of Dental Surgeons*—Meets monthly in Philadelphia. *Pres.* Robert Henry; *Sec.* G. Pettit.

*Pittsburg Dental Society*—Meets second Tuesday of each month. *Pres.* W. F. Fundenburg; *Sec.* M. L. King.

*San Francisco Dental Association*—Meets monthly. *Pres.* C. C. Knowles; *Cor. Sec.* H. E. Knox; *Rec. Sec.* Wm. Dutch.

*St. Louis Dental Society*—Meets monthly. *Pres.* W. H. Morrison; *Sec.* A. H. Fuller.

*Susquehanna Dental Association*—Meets annually. *Pres.* J. N. Johnston; *Rec. Sec.* E. B. Long. Meets November 14, 1877, at Wilkesbarre.

*Society of Dental Surgeons of the City of New York*—Meets semi-monthly in New York. *Pres.* B. F. Franklin; *Rec.* J. S. Latimer; *Cor. Sec.* E. H. Burras.

**Southern Dental Association*—Meets annually. Next meeting at Deer Park, August 14. 1877. *Pre.* J. S. Cobb, Nashville, Tenn.; *Rec. Sec.* E. E. Chisholm, Tuscaloosa, Ala.

*South Carolina State Dental Assocation*—Meets annually. *Pres.* G. F. S. Wright, Columbia; *Sec.* T. F. Cheupein, Charleston; *Cor. Sec* J. S. Thompson, Abbeville. Next meeting at Columbia, the first Tuesday of June, 1877.

*Tennessee Dental Association*—Meets semi-annually. Next meeting at Lookout Mountain June 27, 1877. *Pres.* H. E. Beach; *Sec.* E. S. Chisholm, Tuscaloosa, Ala., *Rec. Sec.* W. H. Morgan; *Cor. Sec.* L. G. Noel.

*Virginia State Dental Association*—Meets in Richmond first Tuesday in November of each year *Pres.* H. M. Grant, Abington; *Sec.* G. F. Keesee, Richmond.

*Washington City Dental Society*—Meets monthly. *Pres.* J. C. Smith; *See.* J. B. TenEyck.

*Wisconsin State Dental Association*—Meets annually. Next meeting at Milwaukee, second Tuesday of July, 1877. *Pres.* R. S. Wells, Sparta; *Sec.* M. T. Moore, La Crosse.

### State and District Dental Societies of State of New York.

*New York State Dental Society*—Meets at Albany on the second Wednesday of July next *Pres.* W. C. Barrett, Warsaw, N. Y.; *Cor. Sec.* S. B. Palmer; *Rec. Sec.* S. S. A. Freeman, Buffalo.

*First District*—Meets second and fourth Wednesday evenings of each month. Annual meeting in New York, first Tuesday in June. *Pres.* J. S. Latimer, New York; *Rec. Sec.* F. M. Odell, New York.

*Second District*—Annual meeting in Brooklyn, first Monday in April. *Pres.* C. D. Cook; *Rec. Sec.* G. L. Mason, Brooklyn; *Cor. Sec.* W. S. Elliott. The meetings are held quarterly, second Thursday in January, April, July and October.

*Third District*—Meets semi-annually on second Tuesday in January and July. Annual meeting at Albany on second Tuesday in January. *Pres.* S. P. Welsh; *Rec. Sec.* H. A. Hall, Troy; *Cor. Sec.* J. A. Perkins, Albany.

*Fourth District*—Meets semi-annually. Meets annually at Saratoga on second Tuesday in June. *Pres.* C. A. Elmore, Ft. Edward; *Rec. Sec.* E. S. Pearsall, Saratoga.

*Fifth District*—Meets semi-annually. Annual meeting at Syracuse, second Tuesday in June. *Pres.* F. D. Nellis, Syracuse; *Rec. Sec.* J. S. Marshall, Syracuse; *Cor. Sec.* A. B. Cowles, Rome.

*Sixth District*—Meets semi-annually. Annual meeting at Binghampton, on second Tuesday in June. *Pres.* Frank B. Darby, Oswego; *Rec. Sec.* F. E. Perry, Norwich; *Cor. Sec.* A. M. Holmes, Morrisville.

*Seventh District*—Meets semi-annually. Annual meeting at Rochester, first Tuesday in June. *Pres.* H. S. Miller; *Rec. Sec.* J. E. Line, Rochester.

*Eighth District*—Meets semi-annually. Annual meeting on second Tuesday in May. *Pres.* G. C. Daboll; *Rec. Sec.* S. A. Freeman; *Cor. Sec.* T. G. Lewis, Buffalo.

*The Seventh and Eighth District Societies*—Hold a union meeting on the last Tuesday of October, alternately at Buffalo and Rochester.

*The Ohio State Board of Dental Examiners* will meet in Columbus on the first Tuesday of December, 1877, at 12 o'clock, M. *Pres.* J. Taft; *Sec.* W. P. Horton.

### EXCHANGES.

ST. LOUIS MEDICAL AND SURGICAL JOURNAL.
BOSTON MEDICAL AND SURGICAL JOURNAL.
THE PACIFIC MEDICAL AND SURGICAL JOURNAL, SAN FRANCISCO.
PHILADELPHIA MEDICAL AND SULGICAL JNURNAL.
CINCINNATI MEDICAL ADVANCE.
CINCINNATI LANCET AND OBSERVER.
DENTAL COSMOS, PHILADELPHIA.
LADIES' REPOSITORY, CINCINNATI.
CHICAGO MEDICAL EXAMINER.
THE DETROIT REVIEW OF MEDICINE AND PHARMACY.
MEDICAL RECORD, N. Y.
NASHVILLE JOURNAL OF MEDICINE AND SURGERY.
AMERICAN JOURNAL OF DENTAL SCIENCE, BALTIMORE.
THE UNITED STATES MEDICAL AND SURGICAL JOURNAL, CHICAGO.
BOSTON JOURNAL OF CHEMISTRY.
JOURNAL OF APPLIED CHEMISTRY.
CANADA JOURNAL OF DENTAL SCIENCE, MONTREAL.
INDIANA JOURNAL OF MEDICINE.
MEDICAL AND SURGICAL REPORTER, SALEM, OREGON.
MISSOURI DENTAL JOURNAL, ST. LOUIS.
ECLECTIC MEDICAL JOURNAL, CINCINNATI.

### HARVARD UNIVERSITY.

### Dental Department.

### BOSTON, MASS. 1877-78.

#### FACULTY.

Charles William Eliot, L.L.D., *President of the University.*
Oliver W. Holmes, M.D., Professor of Anatomy.
Henry J. Bigelow, M.D., Professor of Surgery and Clinical Surgery.
Thomas H. Chandler, D.M.D., Professor of Mechanical Dentistry.
George T. Moffatt, M.D., D.M.D., Professor of Operative Dentistry.
Luther D. Shepard, D.D.S., Adjunct Professor of Operative Dentistry.
Nathaniel W. Hawes, Assistant Professor of Operative Dentistry.
Edward S. Wood, M.D., Assistant Professor of Chemistry.
Henry P. Bowditch, M.D., Assistant Professor of Physiology.
William H. Rollins, D.M.D , Instructor in Dental Pathology.
Charles A. Bracket, D.M.D., Instructor in Dental Therapeutics.
Ira A. Salmon, D.D.S., University Lecturer on Operative Dentistry.
Charles B. Porter, M.D., Demonstrator of Practical Anatomy.
Charles Wilson, D.M.D., Demonstrator in Charge.
George F. Grant, D.M.D., Demonstrator of Mechanical Dentistry.

The plan of study of this School has been radically changed. Instead of the usual oral examinations held at the end of the course, written examinations are had at the end of each year on the subjects pursued during the year, and instead of the customary repetition each year of the lectures and studies of the preceding year, the course is progressive over a period of two years, and the student is required to pass a satisfactory examination in the first year studies before being allowed to proceed to the next. At the end of the course a satisfactory examination must be passed in all the subjects.

The new year begins Sept. 27th, 1877, and ends the last Wednesday in June, 1878. It is divided into two equal terms, with a recess of one week at Christmas.

The regular examinations will be held in the following order, viz.: At the end of the first year, anatomy, including dissection, physiology and general chemistry. A certificate from the demonstrator of anatomy will be required of each student, that he has satisfactorily dissected the three parts of the body.

At the end of the second year, dental pathology, including a knowledge of gestation and diseases of women so far as they affect the mouth and throat, dental materia medica and therapeutics, oral surgery and surgical pathology, operative and mechanical dentistry. The examinations in operative and mechanical dentistry will include actual operations and the preparation of specimens of mechanical dentistry.

Every candidate must be twenty-one years of age, and of good moral character; he must give evidence of having studied medicine or dentistry three full years; he must have spent at least one continuous year at this school, have presented a satisfactory thesis and have passed all the required examinations.

The University Degree, D.M.D. (*Dentariæ Medicinæ Doctor*), is conferred upon those who fulfil the requirements.

Students may be admitted to advanced standing upon passing a satisfactory examination in a majority of the studies already pursued by the class; but no student shall advance with his class or be admitted to advanced standing until he has passed such examination.

The faculty recommend young men who propose to take the degree to spend the whole of the required term of three years of study in the school. But those who wish to spend but two of the three years in the school are earnestly advised to pass their first year of study, before entering, under the direction of a *competent* private instructor.

Unsurpassed facilities for operative and mechanical practice are furnished by the infirmary of the Massachusetts General Hospital which is open throughout the year.

#### FEES.

There are no fees for matriculation, for the diploma, nor for the demonstrators. For the first year a student is a member of the school, the fee is $200; for the second year, $150; for any subsequent year, $50.

For further information address,

**THOS. H. CHANDLER, Dean,**

**222 Tremont Street, Boston, Mass.**

### Ohio College of Dental Surgery.

THE THIRTY-SECOND ANNUAL SESSION OF THE OHIO COLLEGE OF DENTAL SURGERY will begin on the 18th of October, 1877, and continue till March following.

### FACULTY.

JAMES TAYLOR, M. D., D. D. S., Emeritus Institutes of Dental Science.
J. TAFT, D. D. S., Operative Dentistry, Hygiene and Microscopy.
F. BRUNNING, M. D., Pathology and Therapeutics.
J. S. CASSIDY, D. D. S., Chemistry.
WM. CLENDENIN, M. D., Anatomy.
J. L. CILLEY, M. D., Physiology and Histology.
H. M. REID, D. D. S., Clinical Dentistry.
WM. VAN ANTWERP, D. D. S., Mechanical Dentistry and Practical Anatomy.
N. S. HOFF, D. D. S., Demonstrator of Clinical and Mechanical Dentistry.

### SPECIAL PROFESSORS.

GEO. WATT, M. D. D., D. S., Diseases of Women and Children, with reference to Dentistry.
F. H. REHWINKEL, M. D., D. D. S., Oral Surgery.

### CLINICAL LECTURERS AND OPERATORS.

| | | | |
|---|---|---|---|
| W. H. GODDARD, | Louisville, Ky. | J. W. SMITH, | Richmond, Ky. |
| J. K. JAMESON, | Connersville, Ind. | WILL. TAFT, | Cincinnati, O. |
| J. R. CLAYTON, | Shelbyville, Ind. | H. A. SMITH, | Cincinnati, O. |
| S. B. BROWN, | Ft. Wayne, Ind. | C. R. TAFT, | Cincinnati, O |
| J. E. CRAVENS, | Indianapolis, Ind. | FRANK HUNTER, | Cincinnati, O. |
| A. O. RAWLS, | Lexington, Ky. | GEO. W. KEELY, | Oxford, O. |

### FEES.

| | |
|---|---|
| Matriculation Fee, (but once) | $ 5.00 |
| Professors' Tickets for one session | 100.00 |
| Or for Winter and Spring Terms | 130.00 |
| Demonstrator's Ticket, (for Anatomy) | 5.00 |
| Diploma Fee | 30.00 |

### TEXT BOOKS.

Gray's or Wilson's Anatomy; Dalton's, Carpenter's, or Kirk's Physiology; Wagner's Pathology; U. S. Dispensatory; Fowne's, Turner's, or Graham's Chemistry; Watt's Chemical Essays; Taft's Operative Dentistry; Richardson's Mechanical Dentistry; Harris' Principles and Practice of Dental Science; Tome's Dental Surgery; Harris' Dictionary of Dental Surgery.

TERMS OF GRADUATION.—A candidate for graduation must have two full years of pupilage, part of which, at least, should be with a reputable dental practitioner, and good teacher, inclusive of two complete courses of lectures in a Dental College.

A graduate of a respectable Medical College who has had one year's pupilage under a reputable dentist, or a student having had a regular pupilage of two years and four years practice, and otherwise satisfactory, may be admitted to the Senior Class, and to examination for the degree of D. D. S., after attending one full course of lectures.

For further particulars, address the Dean or Secretary by letter.

Students coming to the city should call on the Dean. Good board can be had for $4.00 to $7.00 a week.

F. BRUNNING, SEC., 149 Broadway, Cincinnati, Ohio.
J. TAFT, DEAN, 117 West Fourth St., Cincinnati, Ohio.

*American Dental Association*—Meets on the first Tuesday in Aug., 1877, at Chicago, *Pres.* G. W. Keeley, Oxford, Ohio; *Cor. Sec.* J. H. McQuilien, Philadelphia; *Rec. Sec.* C. Stoddard Smith, Springfield, Ill.

*American Dental Convention*—Meets at Deer Park, Md., second Tuesday in August, 1877. *Pres.* W. H. Atkinson, New York; *Cor. Sec.* G. W. Mills, Baltimore; *Rec. Sec.* A. Tees, Philadelphia, Pa.

LIST OF SOCIETIES REPRESENTED IN THE AMERICAN DENTAL ASSOCIATION:

*American Academy of Dental Surgery*—Meets in New York 187 . *Pres.* Geo. H. Perine; *Rec. Sec.* N. M. Beckwith.

*American Academy of Dental Science*—Meets at Boston, Mass. *Pres.* Dr. D. M. Parker, *Sec.* W. L. Tucker, of Boston: *Cor. Sec.* Geo. T. Moffatt.

*Baltimore Dental Society*—*Pres.* C. T. Brockett; *Rec. Sec.* F. F. Drew.

*Brooklyn Dental Society*—Meets semi-monthly. *Pres.* H. G. Merick; *Rec. Sec.* C. P. Crandell.

*Buffalo Dental Society*—Meets second Monday of each month, at Buffalo. *Pres.* S. A. Freeman; *Rec. Sec.* G. C. Daboll.

*Boston Dental Institute*—Meets first Monday of each month. *Pres.* D. G. Williams, Boston; *Cor. Sec.* D. S. Dickerman.

*California State Dental Association*—Meets First Tuesday in June, 1878. *Pres.* Wm. B. Kingsbury; *Sec.* S. E. Goe.

*Chicago Dental Society*—Meets monthly at Chicago. *Pres.* C. R. E. Koch; *Rec. Sec.* D. B. Freeman.

*Connecticut Valley Dental Association*—Meets semi-annually, second Tuesday of June and October. *Pres.* H. F. Bishop, worcester, Mass.; *Rec. Sec.* C. T. Stockwell, Westfield, Mass. Next meeting in Springfield, Mass., October 17, 1877.

*Connecticut State Dental Society*—*Pres.* E. T. Payne; *Rec. Sec.* Geo. L. Parmelee. Hartford. Semi-annual meeting in October in Hartford.

*Charleston Dental Association*—Meets second Tuesday of every month. *Pres.* T. F. Chupein; *Sec.* and *Treas.* B. A. Muckenfuss.

*Central Pennsylvania Dental Association*—Meets at Huntingdon, Jan., 1878. *Pres.* E. J. Green; *Sec.* W. B. Miller.

*Cincinnati Dental Society*—Meets first and third Tuesday of each month. *Pres.* A. Berry; *Sec.* A. Downing.

*Central Ohio Dental Society*—Meets at Crestline, on the second Monday in April, 1877. *Pres.* M. DeCamp; *Sec.* N. J. Cressinger.

*Dental Society of the State of Maryland and District of Columbia*—Meets annually. Next meeting at Deer Park, on the second Tuesday in August, 1877. *Pres.* R. F. Hunt, of Washington, D. C.; *Sec.* H. C. Thompson.

*Eastern Indiana Dental Association*—Meets semi-annually, first Tuesday in May, and last Tuesday in October; *Pres.* J. W. Ellis, New Castle; *Sec.* M. H. Chappell, Knightstown, Ind. Place of meeting, Cambridge City.

*East Tennessee Dental Association*—Meets annually. Next meeting will be held at Cleveland, Tenn., on the third Wednesday of Sept. 1877. *Pres.* James Carson. *Sec.* J. U. Lee, of Chattanooga.

*Georgia State Dental Society*—July 30th, 1878. *Pres.* M. H. Thomas, Monroe Ga. *Rec. Sec.* J. A. Chopple, La Grange, Ga. *Cor. Sec.* Dr. M. S. Jobson, Perry, Ga.

*Harris Dental Association*—Meets quarterly. Next Meeting at Columbia, Pa., first Thursday in August. *Pres.* J. D. Heiges; *Sec.* W. N. Amer, Lancaster, Pa.

*Hudson Valley Dental Association*—Meets monthly at Troy, N. Y. *Pres.* L. C. Wheeler; *Rec. Sec.* S. G. Andres; *Cor. Sec.* S. D. French.

*Illinois State Dental Society*—Meets annually. *Pres.* C. R. E. Koch; *Sec.* E. D. Swain, Chicago. Next place of meeting, Rockford, second Tuesday in May, 1878.

*Indiana State Dental Society*—Meets next year as Ft. Wayne, on the last Tuesday of June, 1877. *Pres.* Isaac Knapp, Ft. Wayne; *Sec.* J. E. Cravens.

*Iowa State Dental Society*—Meets annually. Next meeting at Iowa City on the second Tuesday of May. *Pres.* G. S. Shattuck, Belle Plane; *Rec. Sec.* E. E. Hughes of Newton; *Cor. Sec.* I. P. Wilson, Burlington.

*Kentucky State Dental Association*—Meets annually first Tuesday in June. Next meeting in Covington. *Pres.* A. H. Smith, of Richmond; *Sec.* F. Peabody of Louisville

*Kansas State Dental Association*—*Pres.* J. D. Patterson; *Sec.* Wm. H. Shulze, of Atchison. Next meeting will be held at Lawreuce, first Tuesday in May, 1878.

*Lake Erie Dental Association*—Meets annually. *Pres.* C. B. Ansart, Oil City, Penn. *Sec.* C. D. Elliot, Franklin, Penn. Next meeting first Tuesday of May, 1876.

*Mad River Dental Association*—Meets at Xenia on the first Tuesday in April and October. *Pres.* R. Corson, Middletown; *Sec.* E. Betty, Xenia.

*Maine Dental Society*—Meets in August, at Thomaston. *Pres.* E. J. Roberts, Augusta, *Sec.* C. E. Hussey, Biddeford.

*Massachusetts Dental Society*—Meets monthly. *Pres.* John T. Codman, Boston; *Rec. Sec.* G. F. Grant, Boston; *Cor. Sec.* T. N. Chandler, Boston.

*Michigan State Dental Association*—Meets annually. Next meeting at Ann Arbor, October 9. *Pres.* R. G. Thomas, Detroit; *Sec.* and *Treas.* E. S. Holmes, Grand Rapids.

*Missippi Valley Dental Association*—Meets annually at Cincinnati, on first Wednesday in March, 1878. *Pres.* A. O. Rawls, Lexington, Ky.; *Rec. Sec.* Wm. Taft, Cincinnati.

*Mississippi State Dental Association*—Will meet third Wednesday of August, 1877, at Canton. *Pres.* J. B. Askew ; *Sec.* A. Riser, Port Gibson.

*Minnesota Dental Association*—Meets annually. Meets May 26, 1878 at Winona. *Pres.* W. F. Lewis; *Sec.* C. M. Bailey, Minneapolis.

*Missouri Dental Association*—Meets annually on the first Tuesday of June. Next meeting at Sweet Springs. *Pres.* G. W. Tindall; *Sec.* W. H. Eames, of St. Louis,

*Missouri Valley Dental Association*—Meets on the fourth Tuesday in July, 1876, in Omaha, Neb. *Pres.* J. W. Chadduck; *Sec.* and *Treas.* F. M. Shrirer, Tabor, Iowa.

*Merrimac Valley Dental Association*—Meets on the 3d and 4th of May, 1877, at Lynn. *Pres.* H. Hill, Manchester, N. H.; *Rec. Sec.* W. E. Riggs, Lawrence, Mass.; *Cor. Sec.* J. H. Kidder, Lawrence, Mass.

*Memphis Dental Society*—Meets on the first Tuesday of each month. *Pres.* W. M. Arrington; *Sec.* V. F. Elliot; *Treas.* C. L. Harris.

*Nashville Dental Association*—Meets on the second Tuesday of each month. *Pres.* R. R. Freeman; *Sec.* L. G. Noel, Nashville.

*Nebraska State Dental Society*—Meets annually. Next meeting July 24th, and 25th. *Pres.* J. S. Charles, Omaha. *Sec.* F. M. Shriver, Glenwood.

*New Jersey State Dental Society*—Meets annually. Next meeting at Long Branch, on the second Tuesday in July, 1877. *Pres.* T. B. Welch, Newark; *Sec.* C. A. Meeker, Newark.

*New Haven Dental Society*—Meets on the first Wednesday of each month at New Haven. *Pres.* J. T. Metcalf; *Rec.* and *Cor. Sec.* C. L. Smith, of New Haven.

*New Orleans Dental Society*—Meets on the first Thursday of each month. *Pres.* W. S. Chandler; *Rec. Sec.* F. J. Knapp.

*Northern Ohio Dental Association*—Meets annually. Next meeting at Elyria, on the second Tuesday in May, 1878. *Pres.* J. W. Lyder, Akron, O. *Sec.* J. F. Siddall, Oberlin.

*Northern Iowa Dental Asssociation*—Meets annually, on the second Tuesday in June, 1873. Next meeting at waterloo. *Pres.* J. N. Abbott Lancaster; *Rec. Sec.* A. V. Eaton. Anamosa; *Cor. Sec.* A. R. Mason, Waterloo.

*North Carolina Dental Association*—Meets annually. Next meeting at Greensboro, second Tuesday of August, 1876. *Pres.* B. F. Arrington; *Sec.* E. L. Hunter.

*North Carolina State Dental Society*—Meets annually. Next meeting at Raleigh, first Wednesday Sept. 1877. *Pres.* V. E. Turner; *Sec.* D. A. Robinson.

*Ohio College Dental Association*—Meets annually, February 25, at Cincinnati. *Pres.* N. G. McKellops, St. Louis, Mo.; *Rec. Sec.* H. A. Smith, of Cincinnati.

*Ohio State Dental Association*—Meets annually at Columbus, first Wednesday of December, 1877. *Pres.* I. Williams; *Rec. Sec.* H. L. Ambler.

*Odontographic Society of Pennsylvania*—Meets on the first Wednesday of each month, at 8 o'clock, P.M., in the Philadelphia Dental College. *Pres.* F. M. Dixon; *Rec. Sec.* E. L. Hewitt.

*Odontological Society of New York City*—Meets the second Tuesday of each month. *Pres.* A. L. Northrup; *Rec. Sec.* Wm. Jarvie, Jr.

*Old Colony Dental Association*—Meets quarterly.* *Pres.* E. L. Hathaway; *Rec. Sec.* L. W. Puffer, North Bridgewater, Mass.

*Ohio Valley Dental Association*—Meets annually in N. E., Ky.; this year in Paris, Ky., Tuesday, September 9th.

*Oregon State Dental Society*—Meets annually. *Pres.* J. R. Cardwell, Portland; *Rec. Sec.* G. N. Chance, Portland.

*Pennsylvania State Dental Society*—Meets next in Minnequa Springs, second Tuesday of July, 1877. *Pres.* J. S. King; *Rec. Sec.* S. H. Guilford; *Cor Sec.* M. H. Webb.

*Pennsylvania Association of Dental Surgeons*—Meets monthly in Philadelphia. *Pres.* Robert Henry; *Sec.* G. Pettit.

*Pittsburg Dental Society*—Meets second Tuesday of each month. *Pres.* W. F. Fundenburg; *Sec.* M. L. King.

*San Francisco Dental Association*—Meets monthly. *Pres.* Alex. Warner; *Vice Pres.* R. F. Rall; *Cor. Sec.* S. E. Goe; *Rec. Sec.* L. L. Dunbar.

*St. Louis Dental Society*—Meets monthly. *Pres.* W. H. Morrison; *Sec.* A. H. Fuller.

*Susquehanna Dental Association*—Meets annually. *Pres.* J. N. Johnston; *Rec. Sec.* E. B. Long. Meets November 14, 1877, at Wilkesbarre.

*Society of Dental Surgeons of the City of New York*—Meets semi-monthly in New York. *Pres.* B. F. Franklin; *Rec.* J. S. Latimer; *Cor. Sec.* E. H. Burras.

**Southern Dental Association*—Meets annually. Next meeting at Deer Park, August 14. 1877. *Pre.* J. S. Cobb, Nashville, Tenn.; *Rec. Sec.* E. E. Chisholm, Tuscaloosa, Ala.

*South Carolina State Dental Assocation*—Meets annually. *Pres.* G. F. S. Wright, Columbia; *Sec.* T. F. Cheupein, Charleston; *Cor. Sec* J. S. Thompson, Abbeville. Next meeting at Columbia, the first Tuesday of June, 1877.

*Tennessee Dental Association*—Meets semi-annually. Next meeting at Lookout Mountain June 27, 1877. *Pres.* H. E. Beach; *Sec.* E. S. Chisholm, Tuscaloosa, Ala., *Rec. Sec.* W. H. Morgan; *Cor. Sec.* L. G. Noel.

*Virginia State Dental Association*—Meets in Richmond first Tuesday in November of each year *Pres.* H. M. Grant, Abington; *Sec.* G. F. Keesee, Richmond.

*Washington City Dental Society*—Meets monthly. *Pres.* J. C. Smith; *See.* J. B. TenEyck.

*Wisconsin State Dental Association*—Meets annually. Next meeting at Milwaukee, second Tuesday of July, 1877. *Pres.* R. S. Wells, Sparta; *Sec.* M. T. Moore, La Crosse.

### State and District Dental Societies of State of New York.

*New York State Dental Society*—Meets at Albany on the second Wednesday of July next *Pres.* W. C. Barrett, Warsaw, N. Y.; *Cor. Sec.* S. B. Palmer; *Rec. Sec.* S. S. A. Freeman, Buffalo.

*First District*—Meets second and fourth Wednesday evenings of each month. Annual meeting in New York, first Tuesday in June. *Pres.* J. S. Latimer, New York; *Rec. Sec.* F. M. Odell, New York.

*Second District*—Annual meeting in Brooklyn, first Monday in April. *Pres.* C. D. Cook; *Rec. Sec.* G. L. Mason, Brooklyn; *Cor. Sec.* W. S. Elliott. The meetings are held quarterly, second Thursday in January, April, July and October.

*Third District*—Meets semi-annually on second Tuesday in January and July. Annual meeting at Albany on second Tuesday in January. *Pres.* S. P. Welsh; *Rec. Sec.* H. A. Hall, Troy; *Cor. Sec.* J. A. Perkins, Albany.

*Fourth District*—Meets semi-annually. Meets annually at Saratoga on second Tuesday in June. *Pres.* C. A. Elmore, Ft. Edward; *Rec. Sec.* E. S. Pearsall, Saratoga.

*Fifth District*—Meets semi-annually. Annual meeting at Syracuse, second Tuesday in June. *Pres.* F. D. Nellis, Syracuse; *Rec. Sec.* J. S. Marshall, Syracuse; *Cor. Sec.* A. B. Cowles, Rome.

*Sixth District*—Meets semi-annually. Annual meeting at Binghampton, on second Tuesday in June. *Pres.* Frank B. Darby, Oswego; *Rec. Sec.* F. E. Perry, Norwich *Cor. Sec.* A. M. Holmes, Morrisville.

*Seventh District*—Meets semi-annually. Annual meeting at Rochester, first Tuesday in June. *Pres.* H. S. Miller; *Rec. Sec.* J. E. Line, Rochester.

*Eighth District*—Meets semi-annually. Annual meeting on second Tuesday in May. *Pres.* G. C. Daboll; *Rec. Sec.* S. A. Freeman; *Cor. Sec.* T. G. Lewis. Buffalo.

*The Seventh and Eighth District Societies*—Hold a union meeting on the last Tuesday of October, alternately at Buffalo and Rochester.

*The Ohio State Board of Dental Examiners* will meet in Columbus on the first Tuesday of December, 1877, at 12 o'clock, M. *Pres.* J. Taft; *Sec.* W. P. Horton.

### EXCHANGES.

ST. LOUIS MEDICAL AND SURGICAL JOURNAL.
BOSTON MEDICAL AND SURGICAL JOURNAL.
THE PACIFIC MEDICAL AND SURGICAL JOURNAL, SAN FRANCISCO.
PHILADELPHIA MEDICAL AND SULGICAL JNURNAL.
CINCINNATI MEDICAL ADVANCE.
CINCINNATI LANCET AND OBSERVER.
DENTAL COSMOS, PHILADELPHIA.
LADIES' REPOSITORY, CINCINNATI.
CHICAGO MEDICAL EXAMINER.
THE DETROIT REVIEW OF MEDICINE AND PHARMACY.
MEDICAL RECORD, N. Y.
NASHVILLE JOURNAL OF MEDICINE AND SURGERY.
AMERICAN JOURNAL OF DENTAL SCIENCE, BALTIMORE.
THE UNITED STATES MEDICAL AND SURGICAL JOURNAL, CHICAGO.
BOSTON JOURNAL OF CHEMISTRY.
JOURNAL OF APPLIED CHEMISTRY.
CANADA JOURNAL OF DENTAL SCIENCE, MONTREAL.
INDIANA JOURNAL OF MEDICINE.
MEDICAL AND SURGICAL REPORTER, SALEM, OREGON.
MISSOURI DENTAL JOURNAL. ST. LOUIS.
ECLECTIC MEDICAL JOURNAL, CINCINNATI.
THE SANITARAN, NEW YORK CITY.

### HARVARD UNIVERSITY.

### Dental Department.

### BOSTON, MASS. 1877-87.

**FACULTY.**

Charles William Eliot, L.L.D., *President of the University.*
Oliver W. Holmes, M.D., Professor of Anatomy.
Henry J. Bigelow, M.D., Professor of Surgery and Clinical Surgery.
Thomas H. Chandler, D.M.D., Professor of Mechanical Dentistry.
George T. Moffatt, M.D., D.M.D., Professor of Operative Dentistry.
Luther D. Shepard, D.D.S., Adjunct Professor of Operative Dentistry.
Nathaniel W. Hawes, Assistant Professor of Operative Dentistry.
Edward S. Wood, M.D., Assistant Professor of Chemistry.
Henry P. Bowditch, M.D., Assistant Professor of Physiology.
William H. Rollins, D.M.D , Instructor in Dental Pathology.
Charles A. Bracket, D.M.D., Instructor in Dental Therapeutics.
Ira A. Salmon, D.D.S., University Lecturer on Operative Dentistry.
Charles B. Porter, M.D., Demonstrator of Practical Anatomy.
Charles Wilson, D.M.D., Demonstrator in Charge.
George F. Grant, D.M.D., Demonstrator of Mechanical Dentistry.

The plan of study of this School has been radically changed. Instead of the usual oral examinations held at the end of the course, written examinations are had at the end of each year on the subjects pursued during the year, and instead of the customary repetition each year of the lectures and studies of the preceding year, the course is progressive over a period of two years, and the student is required to pass a satisfactory examination in the first year studies before being allowed to proceed to the next. At the end of the course a satisfactory examination must be passed in all the subjects.

The new year begins Sept. 27th, 1877, and ends the last Wednesday in June, 1878. It is divided into two equal terms, with a recess of one week at Christmas.

The regular examinations will be held in the following order, viz.: At the end of the first year, anatomy, including dissection, physiology and general chemistry. A certificate from the demonstrator of anatomy will be required of each student, that he has satisfactorily dissected the three parts of the body.

At the end of the second year, dental pathology, including a knowledge of gestation and diseases of women so far as they affect the mouth and throat, dental materia medica and therapeutics, oral surgery and surgical pathology, operative and mechanical dentistry. The examinations in operative and mechanical dentistry will include actual operations and the preparation of specimens of mechanical dentistry.

Every candidate must be twenty-one years of age, and of good moral character; he must give evidence of having studied medicine or dentistry three full years; he must have spent at least one continuous year at this school, have presented a satisfactory thesis and have passed all the required examinations.

The University Degree, D.M.D. (*Dentariæ Medicinæ Doctor*), is conferred upon those who fulfil the requirements.

Students may be admitted to advanced standing upon passing a satisfactory examination in a majority of the studies already pursued by the class; but no student shall advance with his class or be admitted to advanced standing until he has passed such examination.

The faculty recommend young men who propose to take the degree to spend the whole of the required term of three years of study in the school. But those who wish to spend but two of the three years in the school are earnestly advised to pass their first year of study, before entering, under the direction of a *competent* private instructor.

Unsurpassed facilities for operative and mechanical practice are furnished by the infirmary of the Massachusetts General Hospital which is open throughout the year.

**FEES.**

There are no fees for matriculation, for the diploma, nor for the demonstrators. For the first year a student is a member of the school, the fee is $200; for the second year, $150; for any subsequent year, $50.

For further information address,

**THOS. H. CHANDLER, Dean,**

**222 Tremont Street, Boston, Mass.**

Feb-1y.

### Ohio College of Dental Surgery.

THE THIRTY-SECOND ANNUAL SESSION OF THE OHIO COLLEGE OF DENTAL SURGERY will begin on the 18th of October, 1877, and continue till March following.

### FACULTY.

JAMES TAYLOR, M. D., D. D. S., Emeritus Institutes of Dental Science.
J. TAFT, D. D. S., Operative Dentistry, Hygiene and Microscopy.
F. BRUNNING, M. D., Pathology and Therapeutics.
J. S. CASSIDY, D. D. S., Chemistry.
WM. CLENDENIN, M. D., Anatomy.
J. L. CILLEY, M. D., Physiology and Histology.
H. M. REID, D. D. S., Clinical Dentistry.
WM. VAN ANTWERP, D. D. S., Mechanical Dentistry and Practical Anatomy.
N. S. HOFF, D. D. S., Demonstrator of Clinical and Mechanical Dentistry.

### SPECIAL PROFESSORS.

GEO. WATT, M. D. D., D. S., Diseases of Women and Children, with reference to Dentistry.
F. H. REHWINKEL, M. D., D. D. S., Oral Surgery.

### CLINICAL LECTURERS AND OPERATORS.

| | | | |
|---|---|---|---|
| W. H. GODDARD, | Louisville, Ky. | J. W. SMITH, | Richmond, Ky. |
| J. K. JAMESON, | Connersville, Ind. | WILL. TAFT, | Cincinnati, O. |
| J. R. CLAYTON, | Shelbyville, Ind. | H. A. SMITH, | Cincinnati, O. |
| S. B. BROWN, | Ft. Wayne, Ind. | C. R. TAFT, | Cincinnati, O |
| J. E. CRAVENS, | Indianapolis, Ind. | FRANK HUNTER, | Cincinnati, O. |
| A. O. RAWLS, | Lexington, Ky. | GEO. W. KEELY, | Oxford, O. |

### FEES.

| | |
|---|---|
| Matriculation Fee, (but once). ................. | $ 5.00 |
| Professors' Tickets for one session........... | 100.00 |
| Or for Winter and Spring Terms............ .. | 130.00 |
| Demonstrator's Ticket, (for Anatomy)..... | 5.00 |
| Diploma Fee......................... ............ | 30.00 |

### TEXT BOOKS.

Gray's or Wilson's Anatomy; Dalton's, Carpenter's, or Kirk's Physiology; Wagner's Pathology; U. S. Dispensatory; Fowne's, Turner's, or Graham's Chemistry; Watt's Chemical Essays; Taft's Operative Dentistry; Richardson's Mechanical Dentistry; Harris' Principles and Practice of Dental Science; Tome's Dental Surgery; Harris' Dictionary of Dental Surgery.

TERMS OF GRADUATION.--A candidate for graduation must have two full years of pupilage, part of which, at least, should be with a reputable dental practitioner, and good teacher, inclusive of two complete courses of lectures in a Dental College.

A graduate of a respectable Medical College who has had one year's pupilage under a reputable dentist, or a student having had a regular pupilage of two years and four years practice, and otherwise satisfactory, may be admitted to the Senior Class, and to examination for the degree of D. D. S., after attending one full course of lectures.

For further particulars, address the Dean or Secretary by letter.

Students coming to the city should call on the Dean. Good board can be had for $4.00 to $7.00 a week.

F. BRUNNING, SEC., 149 Broadway, Cincinnati, Ohio.
J. TAFT, DEAN, 117 West Fourth St., Cincinnati, Ohio.

June, 6m,

### The Dental College

OF THE

### UNIVERSITY of MICHIGAN

The SECOND ANNUAL SESSION of this Institution will commence on the 1st of October and close on the last Wednesday of March, thus making a course of six months. The regular course of instruction commences at once and will proceed through the term with the customary holiday vacation.

The students in this department will receive instructions in Anatomy, Physiology, Pathology, Chemistry, Materia Medica, Therapeutics and Surgery, from the professors of their respective branches in the *Department of Medicine* and Surgery of the University, when lectures commence and continue the same as with the Dental College.

### FACULTY OF THE DENTAL DEPARTMENT.

J. B. ANGELL, LL. D., President.

J. TAFT, D. D. S., Professor of Principles aud Practice of Operative Dentistry.

J. A. WATLING, D. D, S., Professor of Clinical and Mechanical Dentistry.

W. H. JACKSON, Demonstrator of Mechanical Dentistry.

It is desirable that students should be present at the opening of the session, as the regular course of instruction begins at that time. Seats in the lecture-room are assigned by selection to students in the order of registration on the steward's books; and each student is expected to occupy during the session such seat as he may select. Students on arriving in Ann Arbor should call at the steward's office.

### CONDITION OF GRADUATION.

The candidate must be twenty-one years of age. He must furnish evidence of good moral character.

He must devote three years in the study of his profession, in connection with attendance upon a course of medical lectures. He must attend two full courses of lectures in the Dental College, or one course elsewhere and the last one here, and we recommend that he attend three courses regularly.

He must sustain an examination satisfactory to the Faculty in all the branches taught.

A graduate of the Medical College may enter the senior class, and, if found qualified, may graduate after one year has been devoted to the study of dentistry.

FEES AND EXPENSES.—The fees which must be paid in advance, are as follows:

MATRICULATION FEE.—Residents of Michigan, $10.00; non-residents, $15.00.

ANNUAL DUES.—Residents of Michigan, $15.00; non-residents, $20.00.

GRADUATION FEE.—For all alike $5.00. The admission fee is paid, but once, and entitles the students to the privileges of permanent membership in any department of the University. The annual tax is paid the first year and every year after.

For further particulars address the Dean of the Dental College, Ann Arbor, Michigan.

tf June, 6m. J. TAFT, Dean.

### Ohio College of Dental Surgery.

THE THIRTY-SECOND ANNUAL SESSION OF THE OHIO COLLEGE OF DENTAL SURGERY will begin on the 18th of October, 1877, and continue till March following.

### FACULTY.

JAMES TAYLOR, M. D., D. D. S., Emeritus Institutes of Dental Science.
J. TAFT, D. D. S., Operative Dentistry, Hygiene and Microscopy.
F. BRUNNING, M. D., Pathology and Therapeutics.
J. S. CASSIDY, D. D. S., Chemistry.
WM. CLENDENIN, M. D., Anatomy.
J. L. CILLEY, M. D., Physiology and Histology.
H. M. REID, D. D. S., Clinical Dentistry.
WM. VAN ANTWERP, D. D. S., Mechanical Dentistry and Practical Anatomy.
N. S. HOFF, D. D. S., Demonstrator of Clinical and Mechanical Dentistry.

### SPECIAL PROFESSORS.

GEO. WATT, M. D. D., D. S., Diseases of Women and Children, with reference to Dentistry.
F. H. REHWINKEL, M. D., D. D. S., Oral Surgery.

### CLINICAL LECTURERS AND OPERATORS.

| | | | |
|---|---|---|---|
| W. H. GODDARD, | Louisville, Ky. | J. W. SMITH, | Richmond, Ky. |
| J. K. JAMESON, | Connersville, Ind. | WILL. TAFT, | Cincinnati, O. |
| J. R. CLAYTON, | Shelbyville, Ind. | H. A. SMITH, | Cincinnati, O. |
| S. B. BROWN, | Ft. Wayne, Ind. | C. R. TAFT, | Cincinnati, O |
| J. E. CRAVENS, | Indianapolis, Ind. | FRANK HUNTER, | Cincinnati, O. |
| A. O. RAWLS, | Lexington, Ky. | GEO. W. KEELY, | Oxford, O. |

### FEES.

| | |
|---|---|
| Matriculation Fee, (but once) | $ 5.00 |
| Professors' Tickets for one session | 100.00 |
| Or for Winter and Spring Terms | 130.00 |
| Demonstrator's Ticket, (for Anatomy) | 5.00 |
| Diploma Fee | 30.00 |

### TEXT BOOKS.

Gray's or Wilson's Anatomy; Dalton's, Carpenter's, or Kirk's Physiology; Wagner's Pathology; U. S. Dispensatory; Fowne's, Turner's, or Graham's Chemistry; Watt's Chemical Essays; Taft's Operative Dentistry; Richardson's Mechanical Dentistry; Harris' Principles and Practice of Dental Science; Tome's Dental Surgery; Harris' Dictionary of Dental Surgery.

TERMS OF GRADUATION.—A candidate for graduation must have two full years of pupilage, part of which, at least, should be with a reputable dental practitioner, and good teacher, inclusive of two complete courses of lectures in a Dental College.

A graduate of a respectable Medical College who has had one year's pupilage under a reputable dentist, or a student having had a regular pupilage of two years and four years practice, and otherwise satisfactory, may be admitted to the Senior Class, and to examination for the degree of D. D. S., after attending one full course of lectures.

For further particulars, address the Dean or Secretary by letter.

Students coming to the city should call on the Dean. Good board can be had for $4.00 to $7.00 a week.

F. BRUNNING, SEC., 149 Broadway, Cincinnati, Ohio.
J. TAFT, DEAN, 117 West Fourth St., Cincinnati, Ohio.

June, 6m,

*American Dental Association*—Meets on the first Tuesday in Aug., 1878, at Niagara Falls, *Pres.* F. H. Rehwinkel, Chillicothe, Ohio; *Cor. Sec.* J. H. McQuilien, Philadelphia; *Rec. Sec.* M. S. Dean, Chicago.

*American Dental Convention*—Meets at Niagara Falls, first Tuesday in August, 1878, *Pres.* J. H. Taft, Cincinnati, Ohio *Rec. Sec.* A. Tees, Philadelphia, Pa.

LIST OF SOCIETIES REPRESENTED IN THE AMERICAN DENTAL ASSOCIATION:

*American Academy of Dental Surgery*—Meets in New York 1878. *Pres.* Geo. H. Perine; *Rec. Sec.* N. M. Beckwith.

*American Academy of Dental Science*—Meets at Boston, Mass. *Pres.* Dr. D. M. Parker, *Sec.* W. L. Tucker, of Boston: *Cor. Sec.* Geo. T. Moffatt.

*Baltimore Dental Society*—*Pres.* C. T. Brockett; *Rec. Sec.* F. F. Drew.

*Brooklyn Dental Society*—Meets semi-monthly. *Pres.* H. G. Merick; *Rec. Sec.* C. P. Crandell.

*Buffalo Dental Society*—Meets second Monday of each month, at Buffalo. *Pres.* S. A. Freeman; *Rec. Sec.* G. C. Daboll.

*Boston Dental Institute*—Meets first Monday of each month. *Pres.* D. G. Williams, Boston; *Cor. Sec.* D. S. Dickerman.

*California State Dental Association*—Meets First Tuesday in June, 1878. *Pres.* Wm. B. Kingsbury; *Sec.* H. J. Plainteaux.

*Chicago Dental Society*—Meets monthly at Chicago. *Pres.* C. R. E. Koch; *Rec. Sec.* D. B. Freeman.

*Connecticut Valley Dental Association*—Meets semi-annually, second Tuesday of June and October. *Pres.* H. F. Bishop, worcester, Mass.; *Rec. Sec.* C. T. Stockwell, Westfield, Mass. Next meeting in Springfield, Mass., October 17, 1877.

*Connecticut State Dental Society*—*Pres.* E. T. Payne; *Rec. Sec.* Geo. L. Parmelee. Hartford. Semi-annual meeting in October in Hartford.

*Charleston Dental Association*—Meets second Tuesday of every month. *Pres.* T. F. Chupein; *Sec.* and *Treas.* B. A. Muckenfuss.

*Central Pennsylvania Dental Association*—Meets at Huntingdon, Jan.. 1878. *Pres.* E. J. Green; *Sec.* W. B. Miller.

*Cincinnati Dental Society*—Meets first and third Tuesday of each month. *Pres.* A. Berry; *Sec.* A. Downing.

*Central Ohio Dental Society*—Meets at Crestline, on the second Monday in April, 1877. *Pres.* M. DeCamp; *Sec.* N. J. Cressinger.

*Dental Society of the State of Maryland and District of Columbia*—Meets annually. Next meeting at Deer Park, on the second Tuesday in August, 1877. *Pres.* R. F. Hunt, of Washington, D. C.; *Sec.* H. C. Thompson.

*Eastern Indiana Dental Association*—Meets semi-annually, first Tuesday in May, and last Tuesday in October; *Pres.* J. W. Ellis, New Castle; *Sec.* M. H. Chappell, Knightstown, Ind. Place of meeting, Cambridge City.

*East Tennessee Dental Association*—Meets annually. Next meeting will be held at Cleveland, Tenn., on the third Wednesday of Sept. 1877. *Pres.* James Carson. *Sec.* J. U. Lee, of Chattanooga.

*Georgia State Dental Society*—July 30th, 1878. *Pres.* M. H. Thomas, Monroe Ga. *Rec. Sec.* J. A. Chopple, La Grange, Ga. *Cor. Sec.* Dr. M. S. Jobson, Perry, Ga.

*Harris Dental Association*—Meets quarterly. Next Meeting at Columbia, Pa., first Thursday in August. *Pres.* J. D. Heiges; *Sec.* W. N. Amer, Lancaster, Pa.

*Hudson Valley Dental Association*—Meets monthly at Troy, N. Y. *Pres.* L. C. Wheeler; *Rec. Sec.* S. G. Andres; *Cor. Sec.* S. D. French.

*Illinois State Dental Society*—Meets annually. *Pres.* C. R. E. Koch, Chicago; *Sec.* E. D. Swain, Chicago. Next place of meeting, Rockford, second Tuesday in May, 1878.

*Indiana State Dental Society*—Meets next year at Indianapolis, on the last Tuesday of June, 1878. *Pres.* Dr. T. S. Hacker, Ft. Wayne; *Sec.* J. E. Cravens.

*Iowa State Dental Society*—Meets annually. Next meeting at Iowa City on the second Tuesday of May. *Pres.* G. S. Shattuck, Belle Plane; *Rec. Sec.* E. E. Hughes of Newton; *Cor. Sec.* I. P. Wilson, Burlington.

*Kentucky State Dental Association*—Meets annually first Tuesday in June. Next meeting in Covington. *Pres.* A. H. Smith, of Richmond; *Sec.* F. Peabody of Louisville.

*Kansas State Dental Association*—*Pres.* J. D. Patterson; *Sec.* Wm. H. Shulzc, of Atchison. Next meeting will be held at Lawreuce, first Tuesday in May, 1878.

*Lake Erie Dental Association*—Meets annually. *Pres.* C. B. Ansart, Oil City, Penn. *Sec.* C. D. Elliot, Franklin, Penn. Next meeting first Tuesday of May, 1876.

*Mad River Dental Association*—Meets at Xenia on the first Tuesday in April and October. *Pres.* R. Corson, Middletown; *Sec.* E. Betty, Xenia.

*Maine Dental Society*—Meets in August, at Thomaston. *Pres.* E. J. Roberts, Augusta, *Sec.* C. E. Hussey, Biddeford.

*Massachusetts Dental Society*—Meets monthly. *Pres.* John T. Codman, Boston; *Rec. Sec.* G. F. Grant, Boston; *Cor. Sec.* T. N. Chandler, Boston.

*Michigan State Dental Association*—Meets annually. Next meeting at Ann Arbor, October 9. *Pres.* R. G. Thomas, Detroit; *Sec.* and *Treas.* E. S. Holmes, Grand Rapids.

*Missippi Valley Dental Association*—Meets annually at Cincinnati, on first Wednesday in March, 1878. *Pres.* A. O. Rawls, Lexington, Ky.; *Rec. Sec.* Wm. Taft, Cincinnati.

*Mississippi State Dental Association*—Will meet third Wednesday of August, 1878, at Winona. *Pres.* A. H. Hilgheim, Jackson; *Sec.* M. C. Maachall.
*Minnesota Dental Association*—Meets annually. Meets May 26, 1878 at Winona. *Pres.* W. F. Lewis; *Sec.* C. M. Bailey, Minneapolis.
*Missouri Dental Association*—Meets annually on the first Tuesday of June. Next meeting at Sweet Springs. *Pres.* G. W. Tindall; *Sec.* W. H. Eames, of St. Louis,
*Missouri Valley Dental Association*—Meets on the fourth Tuesday in July, 1876, in Omaha, Neb. *Pres.* J. W. Chadduck; *Sec.* and *Treas.* F. M. Shrirer, Tabor, Iowa.
*Merrimac Valley Dental Association*—Meets on the 3d and 4th of May, 1877, at Lynn. *Pres.* H. Hill, Manchester, N. H.; *Rec. Sec.* W. E. Riggs, Lawrence, Mass.; *Cor. Sec.* J. H. Kidder, Lawrence, Mass.
*Memphis Dental Society*—Meets on the first Tuesday of each month. *Pres.* W. M. Arrington; *Sec.* V. F. Elliot; *Treas.* C. L. Harris.
*Nashville Dental Association*—Meets on the second Tuesday of each month. *Pres.* R. R. Freeman; *Sec.* L. G. Noel, Nashville.
*Nebraska State Dental Society*—Meets annually. Next meeting July 23rd,at Fremont, *Pres.* S. H. King. Lincoln. *Sec.* W. F. Roseman, Fremont.
*New Jersey State Dental Society*—Meets annually. Next meeting at Long Branch, on the second Tuesday in July, 1877. *Pres.* T. B. Welch, Newark; *Sec.* C. A. Meeker, Newark.
*New Haven Dental Society*—Meets on the first Wednesday of each month at New Haven. *Pres.* J. T. Metcalf; *Rec.* and *Cor. Sec.* C. L. Smith, of New Haven.
*New Orleans Dental Society*—Meets on the first Thursday of each month. *Pres.* W. S. Chandler; *Rec. Sec.* F. J. Knapp.
*Northern Ohio Dental Association*—Meets annually. Next meeting at Elyria, on the second Tuesday in May, 1878. *Pres.* J. W. Lyder, Akron, O. *Sec.* J. F. Siddall, Oberlin.
*Northern Iowa Dental Asssociation*—Meets annually, on the second Tuesday in June, 1873. Next meeting at waterloo. *Pres.* J. N. Abbott Lancaster; *Rec. Sec.* A. V. Eaton. Anamosa; *Cor. Sec.* A. R. Mason, Waterloo.
*North Carolina Dental Association*—Meets annually. Next meeting at Raleigh, first Tuesday in Sept. 1877. *Pres.* V. E. Turner; *Sec.* D. A. Robinson.
*North Carolina State Dental Society*—Meets annually. Next meeting at Raleigh, first Wednesday Sept. 1877. *Pres.* V. E. Turner; *Sec.* D. A. Robinson.
*Ohio College Dental Association*—Meets annually, February 25, at Cincinnati. *Pres.* N. G. McKellops, St. Louis, Mo.; *Rec. Sec.* H. A. Smith, of Cincinnati.
*Ohio State Dental Association*—Meets annually at Columbus, first Wednesday of December, 1877. *Pres.* I. Williams; *Rec. Sec.* H. L. Ambler.
*Odon ographic Society of Pennsylvania*—Meets on the first Wednesday of each month, at 8 o'clock, P.M., in the Philadelphia Dental College. *Pres.* F. M. Dixon; *Rec. Sec.* E. L. Hewitt.
*Odontological Society of New York City*—Meets the second Tuesday of each month. *Pres.* A. L. Northrup; *Rec. Sec.* Wm. Jarvie, Jr.
*Old Colony Dental Association*—Meets quarterly.* *Pres.* E. L. Hathaway; *Rec. Sec.* I. W. Puffer, North Bridgewater, Mass.
*Ohio Valley Dental Association*—Meets annually in N. E., Ky.; this year in Paris, Ky., Tuesday, September 9th.
*Oregon State Dental Society*—Meets annually. *Pres.* J. R. Cardwell, Portland; *Rec. Sec.* G. N. Chance, Portland.
*Pennsylvania State Dental Society*—Meets next in Minnequa Springs, second Tuesday of July, 1877. *Pres.* J. S. King; *Rec. Sec.* S. H. Guilford; *Cor Sec.* M. H. Webb.
*Pennsylvania Association of Dental Surgeons*—Meets monthly in Philadelphia. *Pres.* Robert Henry; *Sec.* G. Pettit.
*Pittsburg Dental Society*—Meets second Tuesday of each month. *Pres.* H. W. Arthur, of Alleghaney; *Sec.* E. C. Diehl, Pitttbuagh.
*San Francisco Dental Association*—Meets monthly. *Pres.* Alex. Warner; *Vice Pres.* R. F. Rall; *Cor. Sec.* S. E. Goe; *Rec. Sec.* L. L. Dunbar.
*St. Louis Dental Society*—Meets monthly. *Pres.* W. H. Morrison; *Sec.* A. H. Fuller.
*Susquehanna Dental Association*—Meets annually. *Pres.* J. N. Johnston; *Rec. Sec.* E. B. Long. Meets November 14, 1877, at Wilkesbarre.
*Society of Dental Surgeons of the City of New York*—Meets semi-monthly in New York. *Pres.* B. F. Franklin; *Rec.* J. S. Latimer; *Cor. Sec.* E. H. Burras.
**Southern Dental Association*—Meets annually. Next meeting at Deer Park, August 14. 1877. *Pre.* J. S. Cobb, Nashville, Tenn.; *Rec. Sec.* E. E. Chisholm, Tuscaloosa, Ala.
*South Carolina State Dental Assocation*—Meets annually. *Pres.* G. F. S. Wright, Columbia; *Sec.* T. F. Cheupein, Charleston; *Cor. Sec* J. S. Thompson, Abbeville. Next meeting at Columbia, the first Tuesday of June, 1877.
*Tennessee Dental Association*—Meets semi-annually. Next meeting at Lookout Mountain June 27, 1877. *Pres.* H. E. Beach; *Sec.* E. S. Chisholm, Tuscaloosa, Ala., *Rec. Sec.* W. H. Morgan; *Cor. Sec.* L. G. Noel.
*Virginia State Dental Association*—Meets in Richmond December 14. *Pres.* W. W. H. Thackston, Farmville; *Sec.* G. F. Keesee, Richmond.
*Washington City Dental Society*—Meets monthly. *Pres.* J. C. Smith; *Sec.* J. B. TenEyck.
*Wisconsin State Dental Association*—Meets annually. Next meeting at Milwaukee, second Tuesday of July, 1877. *Pres.* R. S. Wells, Sparta; *Sec.* M. T. Moore, La Crosse.

### State and District Dental Societies of State of New York.

*New York State Dental Society*—Meets at Albany on the second Wednesday of July next *Pres.* W. C. Barrett, Warsaw, N. Y.; *Cor. Sec.* S. B. Palmer; *Rec. Sec.* S. S. A. Freeman, Buffalo.

*First District*—Meets second and fourth Wednesday evenings of each month. Annual meeting in New York, first Tuesday in June. *Pres.* J. S. Latimer, New York; *Rec. Sec.* F. M. Odell, New York.

*Second District*—Annual meeting in Brooklyn, first Monday in April. *Pres.* C. D. Cook; *Rec. Sec.* G. L. Mason, Brooklyn; *Cor. Sec.* W. S. Elliott. The meetings are held quarterly, second Thursday in January, April, July and October.

*Third District*—Meets semi-annually on second Tuesday in January and July. Annual meeting at Albany on second Tuesday in January. *Pres.* S. P. Welsh; *Rec. Sec.* H. A. Hall, Troy; *Cor. Sec.* J. A. Perkins, Albany.

*Fourth District*—Meets semi-annually. Meets annually at Saratoga on second Tuesday in June. *Pres.* C. A. Elmore, Ft. Edward; *Rec. Sec.* E. S. Pearsall, Saratoga.

*Fifth District*—Meets semi-annually. Annual meeting at Syracuse, second Tuesday in June. *Pres.* F. D. Nellis, Syracuse; *Rec. Sec.* J. S. Marshall, Syracuse; *Cor. Sec.* A. B. Cowles, Rome.

*Sixth District*—Meets semi-annually. Annual meeting at Binghampton, on second Tuesday in June. *Pres.* Frank B. Darby, Oswego; *Rec. Sec.* F. E. Perry, Norwich *Cor. Sec.* A. M. Holmes, Morrisville.

*Seventh District*—Meets semi-annually. Annual meeting at Rochester, first Tuesday in June. *Pres.* H. S. Miller; *Rec. Sec.* J. E. Line, Rochester.

*Eighth District*—Meets semi-annually. Annual meeting on second Tuesday in May. *Pres.* G. C. Daboll; *Rec. Sec.* S. A. Freeman; *Cor. Sec.* T. G. Lewis, Buffalo.

*The Seventh and Eighth District Societies*—Hold a union meeting on the last Tuesday of October, alternately at Buffalo and Rochester.

*The Ohio State Board of Dental Examiners* will meet in Columbus on the first Tuesday of December, 1877, at 12 o'clock, M. *Pres.* J. Taft; *Sec.* W. P. Horton.

### EXCHANGES.

ST. LOUIS MEDICAL AND SURGICAL JOURNAL.
BOSTON MEDICAL AND SURGICAL JOURNAL.
THE PACIFIC MEDICAL AND SURGICAL JOURNAL, SAN FRANCISCO.
PHILADELPHIA MEDICAL AND SULGICAL JNURNAL.
CINCINNATI MEDICAL ADVANCE.
CINCINNATI LANCET AND OBSERVER.
DENTAL COSMOS, PHILADELPHIA.
LADIES' REPOSITORY, CINCINNATI.
CHICAGO MEDICAL EXAMINER.
THE DETROIT REVIEW OF MEDICINE AND PHARMACY.
MEDICAL RECORD, N. Y.
NASHVILLE JOURNAL OF MEDICINE AND SURGERY.
AMERICAN JOURNAL OF DENTAL SCIENCE, BALTIMORE.
THE UNITED STATES MEDICAL AND SURGICAL JOURNAL, CHICAGO.
BOSTON JOURNAL OF CHEMISTRY.
JOURNAL OF APPLIED CHEMISTRY.
CANADA JOURNAL OF DENTAL SCIENCE, MONTREAL.
INDIANA JOURNAL OF MEDICINE.
MEDICAL AND SURGICAL REPORTER, SALEM, OREGON.
MISSOURI DENTAL JOURNAL. ST. LOUIS.
ECLECTIC MEDICAL JOURNAL, CINCINNATI.
THE SANITARAN, NEW YORK CITY.

### Ohio College of Dental Surgery.

The Thirty-Second Annual Session of the Ohio College of Dental Surgery will begin on the 18th of October, 1877, and continue till March following.

### FACULTY.

James Taylor, M. D., D. D. S., Emeritus Institutes of Dental Science.
J. Taft, D. D. S., Operative Dentistry, Hygiene and Microscopy.
F. Brunning, M. D., Pathology and Therapeutics.
J. S. Cassidy, D. D. S., Chemistry.
Wm. Clendenin, M. D., Anatomy.
J. L. Cilley, M. D., Physiology and Histology.
H. M. Reid, D. D. S., Clinical Dentistry.
Wm. Van Antwerp, D. D. S., Mechanical Dentistry and Practical Anatomy.
N. S. Hoff, D. D. S., Demonstrator of Clinical and Mechanical Dentistry.

### SPECIAL PROFESSORS.

Geo. Watt, M. D. D., D. S., Diseases of Women and Children, with reference to Dentistry.
F. H. Rehwinkel, M. D., D. D. S., Oral Surgery.

### CLINICAL LECTURERS AND OPERATORS.

| | | | |
|---|---|---|---|
| W. H. Goddard, | Louisville, Ky. | J. W. Smith, | Richmond, Ky. |
| J. K. Jameson, | Connersville, Ind. | Will. Taft, | Cincinnati, O. |
| J. R. Clayton, | Shelbyville, Ind. | H. A. Smith, | Cincinnati, O. |
| S. B. Brown, | Ft. Wayne, Ind. | C. R. Taft, | Cincinnati, O |
| J. E. Cravens, | Indianapolis, Ind. | Frank Hunter, | Cincinnati, O. |
| A. O. Rawls, | Lexington, Ky. | Geo. W. Keely, | Oxford, O. |

### FEES.

| | |
|---|---|
| Matriculation Fee, (but once)................... | $ 5.00 |
| Professors' Tickets for one session............ | 100.00 |
| Or for Winter and Spring Terms............ .. | 130.00 |
| Demonstrator's Ticket, (for Anatomy)..... | 5.00 |
| Diploma Fee........................... ............ | 30.00 |

### TEXT BOOKS.

Gray's or Wilson's Anatomy; Dalton's, Carpenter's, or Kirk's Physilogy; Wagner's Pathology; U. S. Dispensatory; Fowne's, Turner's, or Graham's Chemistry; Watt's Chemical Essays; Taft's Operative Dentistry; Richardson's Mechanical Dentistry; Harris' Principles and Practice of Dental Science; Tome's Dental Surgery; Harris' Dictionary of Dental Surgery.

Terms of Graduation.--A candidate for graduation must have two full years of pupilage, part of which, at least, should be with a reputable dental practitioner, and good teacher, inclusive of two complete courses of lectures in a Dental College.

A graduate of a respectable Medical College who has had one year's pupilage under a reputable dentist, or a student having had a regular pupilage of two years and four years practice, and otherwise satisfactory, may be admitted to the Senior Class, and to examination for the degree of D. D. S., after attending one full course of lectures.

For further particulars, address the Dean or Secretary by letter.

Students coming to the city should call on the Dean. Good board can be had for $4.00 to $7.00 a week.

F. BRUNNING, Sec., 149 Broadway, Cincinnati, Ohio.
J. TAFT, Dean, 117 West Fourth St., Cincinnati, Ohio.

June, 6m,

*American Dental Association*—Meets on the first Tuesday in Aug., 1878, at Niagara Falls, *Pres.* F. H. Rehwinkel, Chillicothe, Ohio; *Cor. Sec.* J. H. McQuilien, Philadelphia; *Rec. Sec.* M. S. Dean, Chicago.

*American Dental Convention*—Meets at Niagara Falls, first Tuesday in August, 1878. *Pres.* J. H. Taft, Cincinnati, Ohio *Rec. Sec.* A. Tees, Philadelphia, Pa.

List of Societies Represented in the American Dental Association:

*American Academy of Dental Surgery*—Meets in New York 1878. *Pres.* Geo. H. Perine; *Rec. Sec.* N. M. Beckwith.

*American Academy of Dental Science*—Meets at Boston, Mass. *Pres.* Dr. D. M. Parker, *Sec.* W. L. Tucker, of Boston: *Cor. Sec.* Geo. T. Moffatt.

*Baltimore Dental Society*—*Pres.* C. T. Brockett; *Rec. Sec.* F. F. Drew.

*Brooklyn Dental Society*—Meets semi-monthly. *Pres.* H. G. Merick; *Rec. Sec.* C. P. Crandell.

*Buffalo Dental Society*—Meets second Monday of each month, at Buffalo. *Pres.* S. A. Freeman; *Rec. Sec.* G. C. Daboll.

*Boston Dental Institute*—Meets first Monday of each month. *Pres.* D. G. Williams, Boston; *Cor. Sec.* D. S. Dickerman.

*California State Dental Association*—Meets First Tuesday in June, 1878. *Pres.* Wm. B. Kingsbury; *Sec.* H. J. Plainteaux.

*Chicago Dental Society*—Meets monthly at Chicago. *Pres.* C. R. E. Koch; *Rec. Sec.* D. B. Freeman.

*Connecticut Valley Dental Association*—Meets semi-annually, second Tuesday of June and October. *Pres.* H. F. Bishop, worcester, Mass.; *Rec. Sec.* C. T. Stockwell, Westfield, Mass. Next meeting in Springfield, Mass., October 17, 1877.

*Connecticut State Dental Society*—*Pres.* E. T. Payne; *Rec. Sec.* Geo. L. Parmelee. Hartford. Semi-annual meeting in October in Hartford.

*Charleston Dental Association*—Meets second Tuesday of every month. *Pres.* T. F. Chupein; *Sec.* and *Treas.* B. A. Muckenfuss.

*Central Pennsylvania Dental Association*—Meets at Huntingdon, Jan.. 1878. *Pres.* E. J. Green; *Sec.* W. B. Miller.

*Cincinnati Dental Society*—Meets first and third Tuesday of each month. *Pres.* A. Berry; *Sec.* A. Downing.

*Central Ohio Dental Society*—Meets at Crestline, on the second Monday in April, 1877. *Pres.* M. DeCamp; *Sec.* N. J. Cressinger.

*Dental Society of the State of Maryland and District of Columbia*—Meets annually. Next meeting at Deer Park, on the second Tuesday in August, 1877. *Pres.* R. F. Hunt, of Washington, D. C.; *Sec.* H. C. Thompson.

*Eastern Indiana Dental Association*—Meets semi-annually, first Tuesday in May, and last Tuesday in October; *Pres.* J. W. Ellis, New Castle; *Sec.* M. H. Chappell, Knightstown, Ind. Place of meeting, Cambridge City.

*East Tennessee Dental Association*—Meets annually. Next meeting will be held at Cleveland, Tenn., on the third Wednesday of Sept. 1877. *Pres.* James Carson. *Sec.* J. U. Lee, of Chattanooga.

*Georgia State Dental Society*—July 30th, 1878. *Pres.* M. H. Thomas, Monroe Ga. *Rec. Sec.* J. A. Chopple, La Grange, Ga. *Cor. Sec.* Dr. M. S. Jobson, Perry, Ga.

*Harris Dental Association*—Meets quarterly. Next Meeting at Columbia, Pa., first Thursday in August. *Pres.* J. D. Heiges; *Sec.* W. N. Amer, Lancaster, Pa.

*Hudson Valley Dental Association*—Meets monthly at Troy, N. Y. *Pres.* L. C. Wheeler; *Rec. Sec.* S. G. Andres; *Cor. Sec.* S. D. French.

*Illinois State Dental Society*—Meets annually. *Pres.* C. R. E. Koch, Chicago; *Sec.* E. D. Swain, Chicago. Next place of meeting, Rockford, second Tuesday in May, 1878.

*Indiana State Dental Society*—Meets next year at Indianapolis, on the last Tuesday of June, 1878. *Pres.* Dr. T. S. Hacker, Ft. Wayne; *Sec.* J. E. Cravens.

*Iowa State Dental Society*—Meets annually. Next meeting at Iowa City on the second Tuesday of May. *Pres.* G. S. Shattuck, Belle Plane; *Rec. Sec.* E. E. Hughes of Newton; *Cor. Sec.* I. P. Wilson, Burlington.

*Kentucky State Dental Association*—Meets annually first Tuesday in June. Next meeting in Covington. *Pres.* A. H. Smith, of Richmond; *Sec.* F. Peabody of Louisville.

*Kansas State Dental Association*—*Pres.* J. D. Patterson; *Sec.* Wm. H. Shulze, of Atchison. Next meeting will be held at Lawreuce, first Tuesday in May, 1878.

*Lake Erie Dental Association*—Meets annually. *Pres.* C. B. Ansart, Oil City, Penn. *Sec.* C. D. Elliot, Franklin, Penn. Next meeting first Tuesday of May, 1876.

*Mad River Dental Association*—Meets at Xenia on the first Tuesday in April and October. *Pres.* R. Corson, Middletown; *Sec.* E. Betty, Xenia.

*Maine Dental Society*—Meets in August, at Thomaston. *Pres.* E. J. Roberts, Augusta, *Sec.* C. E. Hussey, Biddeford.

*Massachusetts Dental Society*—Meets monthly. *Pres.* John T. Codman, Boston; *Rec. Sec.* G. F. Grant, Boston; *Cor. Sec.* T. N. Chandler, Boston.

*Michigan State Dental Association*—Meets annually. Next meeting at Ann Arbor, October 9. *Pres.* R. G. Thomas, Detroit; *Sec.* and *Treas.* E. S. Holmes, Grand Rapids.

*Missippi Valley Dental Association*—Meets annually at Cincinnati, on first Wednesday in March, 1878. *Pres.* A. O. Rawls, Lexington, Ky.; *Rec. Sec.* Wm. Taft, Cincinnati.

*Mississippi State Dental Association*—Will meet third Wednesday of August, 1878, at Winona. *Pres.* A. H. Hilzheim, Jackson; *Sec* M. C. Marshall.

*Minnesota Dental Association*—Meets annually. Meets May 26, 1878 at Winona. *Pres.* W. F. Lewis; *Sec.* C. M. Bailey, Minneapolis.

*Missouri Dental Association*—Meets annually on the first Tuesday of June. Next meeting at Sweet Springs. *Pres.* G. W. Tindall; *Sec.* W. H. Eames, of St. Louis,

*Missouri Valley Dental Association*—Meets on the fourth Tuesday in July, 1876, in Omaha, Neb. *Pres.* J. W. Chadduck; *Sec.* and *Treas.* F. M. Shrirer, Tabor, Iowa.

*Merrimac Valley Dental Association*—Meets on the 3d and 4th of May, 1877, at Lynn. *Pres.* H. Hill, Manchester, N. H.; *Rec. Sec.* W. E. Riggs, Lawrence, Mass.; *Cor. Sec.* J. H. Kidder, Lawrence, Mass.

*Memphis Dental Society*—Meets on the first Tuesday of each month. *Pres.* W. M. Arrington; *Sec.* V. F. Elliot; *Treas.* C. L. Harris.

*Nashville Dental Association*—Meets on the second Tuesday of each month. *Pres.* R. R. Freeman; *Sec.* L. G. Noel, Nashville.

*Nebraska State Dental Society*—Meets annually. Next meeting July 23rd, at Fremont. *Pres.* S. H. King. Lincoln. *Sec.* W. F. Roseman, Fremont.

*New Jersey State Dental Society*—Meets annually. Next meeting at Long Branch, on the second Tuesday in July, 1877. *Pres.* T. B. Welch, Newark; *Sec.* C. A. Meeker, Newark.

*New Haven Dental Society*—Meets on the first Wednesday of each month at New Haven. *Pres.* J. T. Metcalf; *Rec.* and *Cor. Sec.* C. L. Smith, of New Haven.

*New Orleans Dental Society*—Meets on the first Thursday of each month. *Pres.* W. S. Chandler; *Rec. Sec.* F. J. Knapp.

*Northern Ohio Dental Association*—Meets annually. Next meeting at Elyria, on the second Tuesday in May, 1878. *Pres.* J. W. Lyder, Akron, O. *Sec.* J. F. Siddall, Oberlin.

*Northern Iowa Dental Association*—Meets annually, on the second Tuesday in June, 1873. Next meeting at waterloo. *Pres.* J. N. Abbott Lancaster; *Rec. Sec.* A. V. Eaton. Anamosa; *Cor. Sec.* A. R. Mason, Waterloo.

*North Carolina Dental Association*—Meets annually. Next meeting at Charlotte first Tuesday in Sept. 1878. *Pres.* J. W. Hunter, Salem; *Sec.* J. H. Crawford, Raleigh.

*North Carolina State Dental Society*—Meets annually. Next meeting at Raleigh, first Wednesday Sept. 1877. *Pres.* V. E. Turner; *Sec.* D. A. Robinson.

*Ohio College Dental Association*—Meets annually, February 25, at Cincinnati. *Pres.* N. G. McKellops, St. Louis, Mo.; *Rec. Sec.* H. A. Smith, of Cincinnati.

*Ohio State Dental Association*—Meets annually at Columbus, first Wednesday of Decembter, 1877. *Pres.* I. Williams; *Rec. Sec.* H. L. Ambler.

*O lon ographic Society of Pennsylvania*—Meets on the first Wednesday of each month, at 8 o'clock, P.M., in the Philadelphia Dental College. *Pres.* F. M. Dixon; *Rec. Sec.* E. L. Hewitt.

*Odontological Society of New York City*—Meets the second Tuesday of each month. *Pres.* A. L. Northrup; *Rec. Sec.* Wm. Jarvie, Jr.

*Old Colony Dental Association*—Meets quarterly.* *Pres.* E. L. Hathaway; *Rec. Sec.* L. W. Puffer, North Bridgewater, Mass.

*Ohio Valley Dental Association*—Meets annually in N. E., Ky.; this year in Paris, Ky., Tuesday, September 9th.

*Oregon State Dental Society*—Meets annually. *Pres.* J. R. Cardwell, Portland; *Rec. Sec.* G. N. Chance, Portland.

*Pennsylvania State Dental Society*—Meets next in Minnequa Springs, second Tuesday of July, 1877. *Pres.* J. S. King; *Rec. Sec.* S. H. Guilford; *Cor Sec.* M. H. Webb.

*Pennsylvania Association of Dental Surgeons*—Meets monthly in Philadelphia. *Pres.* Robert Henry; *Sec.* G. Pettit.

*Pittsburg Dental Society*—Meets second Tuesday of each month. *Pres.* H. W. Arthur, of Alleghaney; *Sec.* E. C. Diehl, Pitttbuagh.

*San Francisco Dental Association*—Meets monthly. *Pres.* Alex. Warner; *Vice Pres.* R. F. Rall; *Cor. Sec.* S. E. Goe; *Rec. Sec.* L. L. Dunbar.

*St. Louis Dental Society*—Meets monthly. *Pres.* W. H. Morrison; *Sec.* A. H. Fuller.

*Susquehanna Dental Association*—Meets annually. *Pres.* J. N. Johnston; *Rec. Sec.* E. B. Long. Meets November 14, 1877, at Wilkesbarre.

*Society of Dental Surgeons of the City of New York*—Meets semi-monthly in New York. *Pres.* B. F. Franklin; *Rec.* J. S. Latimer; *Cor. Sec.* E. H. Burras.

**Southern Dental Association*—Meets annually. Next meeting at Deer Park, August 14. 1877. *Pre.* J. S. Cobb, Nashville, Tenn.; *Rec. Sec.* E. E. Chisholm, Tuscaloosa, Ala.

*South Carolina State Dental Assocation*—Meets annually. *Pres.* G. F. S. Wright, Columbia; *Sec.* T. F. Cheupein, Charleston; *Cor. Sec* J. S. Thompson, Abbeville. Next meeting at Columbia, the first Tuesday of June, 1877.

*Tennessee Dental Association*—Meets semi-annually. Next meeting at Lookout Mountain June 27, 1877. *Pres.* H. E. Beach; *Sec.* E. S. Chisholm, Tuscaloosa, Ala., *Rec. Sec.* W. H. Morgan; *Cor. Sec.* L. G. Noel.

*Virginia State Dental Association*—Meets in Richmond December 14. *Pres.* W. W. H. Thackston, Farmville; *Sec.* G. F. Keesee, Richmond.

*Washington City Dental Society*—Meets monthly. *Pres.* J. C. Smith; *See.* J. B. TenEyck.

*Wisconsin State Dental Association*—Meets annually. Next meeting at Milwaukee, second Tuesday of July, 1877. *Pres.* R. S. Wells, Sparta; *Sec.* M. T. Moore, La Crosse.

### State and District Dental Societies of State of New York.

*New York State Dental Society*—Meets at Albany on the second Wednesday of July next *Pres.* W. C. Barrett, Warsaw, N. Y.; *Cor. Sec.* S. B. Palmer; *Rec. Sec.* S. S. A. Freeman, Buffalo.

*First District*—Meets second and fourth Wednesday evenings of each month. Annual meeting in New York, first Tuesday in June. *Pres.* J. S. Latimer, New York; *Rec. Sec.* F. M. Odell, New York.

*Second District*—Annual meeting in Brooklyn, first Monday in April. *Pres.* C. D. Cook; *Rec. Sec.* G. L. Mason, Brooklyn; *Cor. Sec.* W. S. Elliott. The meetings are held quarterly, second Thursday in January, April, July and October.

*Th rd District*—Meets semi-annually on second Tuesday in January and July. Annual meeting at Albany on second Tuesday in January. *Pres.* S. P. Welsh; *Rec. Sec.* H. A. Hall, Troy; *Cor. Sec.* J. A. Perkins, Albany.

*Fourth District*—Meets semi-annually. Meets annually at Saratoga on second Tuesday in June. *Pres.* C. A. Elmore, Ft. Edward; *Rec. Sec.* E. S. Pearsall, Saratoga.

*Fifth District*—Meets semi-annually. Annual meeting at Syracuse, second Tuesday in June. *Pres.* F. D. Nellis, Syracuse; *Rec. Sec.* J. S. Marshall, Syracuse; *Cor. Sec.* A. B. Cowles, Rome.

*Sixth District*—Meets semi-annually. Annual meeting at Binghampton, on second Tuesday in June. *Pres.* Frank B. Darby, Oswego; *Rec. Sec.* F. E. Perry, Norwich *Cor. Sec.* A. M. Holmes, Morrisville.

*Seventh District*—Meets semi-annually. Annual meeting at Rochester, first Tuesday in June. *Pres.* H. S. Miller; *Rec. Sec.* J. E. Line, Rochester.

*Eighth District*—Meets semi-annually. Annual meeting on second Tuesday in May. *Pres.* G. C. Daboll; *Rec. Sec.* S. A. Freeman; *Cor. Sec.* T. G. Lewis, Buffalo.

*The Sevent and Eighth District Societies*—Hold a union meeting on the last Tuesday of October, alternately at Buffalo and Rochester.

*The Ohio State Board of Dental Examiners* will meet in Columbus on the first Tuesday of December, 1877, at 12 o'clock, M. *Pres.* J. Taft; *Sec.* W. P. Horton.

### EXCHANGES.

ST. LOUIS MEDICAL AND SURGICAL JOURNAL.
BOSTON MEDICAL AND SURGICAL JOURNAL.
THE PACIFIC MEDICAL AND SURGICAL JOURNAL, SAN FRANCISCO.
PHILADELPHIA MEDICAL AND SULGICAL JNURNAL.
CINCINNATI MEDICAL ADVANCE.
CINCINNATI LANCET AND OBSERVER.
DENTAL COSMOS, PHILADELPHIA.
LADIES' REPOSITORY, CINCINNATI.
CHICAGO MEDICAL EXAMINER.
THE DETROIT REVIEW OF MEDICINE AND PHARMACY.
MEDICAL RECORD, N. Y.
NASHVILLE JOURNAL OF MEDICINE AND SURGERY.
AMERICAN JOURNAL OF DENTAL SCIENCE, BALTIMORE.
THE UNITED STATES MEDICAL AND SURGICAL JOURNAL, CHICAGO.
BOSTON JOURNAL OF CHEMISTRY.
JOURNAL OF APPLIED CHEMISTRY.
CANADA JOURNAL OF DENTAL SCIENCE, MONTREAL.
INDIANA JOURNAL OF MEDICINE.
MEDICAL AND SURGICAL REPORTER, SALEM, OREGON.
MISSOURI DENTAL JOURNAL. ST. LOUIS.
ECLECTIC MEDICAL JOURNAL, CINCINNATI.
THE SANITARAN, NEW YORK CITY.

### Ohio College of Dental Surgery.

THE THIRTY-SECOND ANNUAL SESSION OF THE OHIO COLLEGE OF DENTAL SURGERY will begin on the 18th of October, 1877, and continue till March following.

### FACULTY.

JAMES TAYLOR, M. D., D. D. S., Emeritus Institutes of Dental Science.
J. TAFT, D. D. S., Operative Dentistry, Hygiene and Microscopy.
F. BRUNNING, M. D., Pathology and Therapeutics.
J. S. CASSIDY, D. D. S., Chemistry.
WM. CLENDENIN, M. D., Anatomy.
J. L. CILLEY, M. D., Physiology and Histology.
H. M. REID, D. D. S., Clinical Dentistry.
WM. VAN ANTWERP, D. D. S., Mechanical Dentistry and Practical Anatomy.
N. S. HOFF, D. D. S., Demonstrator of Clinical and Mechanical Dentistry.

### SPECIAL PROFESSORS.

GEO. WATT, M. D. D., D. S., Diseases of Women and Children, with reference to Dentistry.
F. H. REHWINKEL, M. D., D. D. S., Oral Surgery.

### CLINICAL LECTURERS AND OPERATORS.

| | | | |
|---|---|---|---|
| W. H. GODDARD, | Louisville, Ky. | J. W. SMITH, | Richmond, Ky. |
| J. K. JAMESON, | Connersville, Ind. | WILL. TAFT, | Cincinnati, O. |
| J. R. CLAYTON, | Shelbyville, Ind. | H. A. SMITH, | Cincinnati, O. |
| S. B. BROWN, | Ft. Wayne, Ind. | C. R. TAFT, | Cincinnati, O |
| J. E. CRAVENS, | Indianapolis, Ind. | FRANK HUNTER, | Cincinnati, O. |
| A. O. RAWLS, | Lexington, Ky. | GEO. W. KEELY, | Oxford, O. |

### FEES.

| | |
|---|---|
| Matriculation Fee, (but once)................... | $ 5.00 |
| Professors' Tickets for one session............ | 100.00 |
| Or for Winter and Spring Terms............ .. | 130.00 |
| Demonstrator's Ticket, (for Anatomy)..... | 5.00 |
| Diploma Fee......................... ............ | 30.00 |

### TEXT BOOKS.

Gray's or Wilson's Anatomy; Dalton's, Carpenter's, or Kirk's Physiology; Wagner's Pathology; U. S. Dispensatory; Fowne's, Turner's, or Graham's Chemistry; Watt's Chemical Essays; Taft's Operative Dentistry; Richardson's Mechanical Dentistry; Harris' Principles and Practice of Dental Science; Tome's Dental Surgery; Harris' Dictionary of Dental Surgery.

TERMS OF GRADUATION.—A candidate for graduation must have two full years of pupilage, part of which, at least, should be with a reputable dental practitioner, and good teacher, inclusive of two complete courses of lectures in a Dental College.

A graduate of a respectable Medical College who has had one year's pupilage under a reputable dentist, or a student having had a regular pupilage of two years and four years practice, and otherwise satisfactory, may be admitted to the Senior Class, and to examination for the degree of D. D. S., after attending one full course of lectures.

For further particulars, address the Dean or Secretary by letter.

Students coming to the city should call on the Dean. Good board can be had for $4.00 to $7.00 a week.

F. BRUNNING, SEC., 149 Broadway, Cincinnati, Ohio.
J. TAFT, DEAN, 117 West Fourth St., Cincinnati, Ohio.

June, 6m,

### HARVARD UNIVERSITY.

### Dental Department.

### BOSTON, MASS. 1877-87.

**FACULTY.**

CHARLES WILLIAM ELIOT, L.L.D., *President of the University.*
OLIVER W. HOLMES, M.D., Professor of Anatomy.
HENRY J. BIGELOW, M.D., Professor of Surgery and Clinical Surgery.
THOMAS H. CHANDLER, D.M.D., Professor of Mechanical Dentistry.
GEORGE T. MOFFATT, M.D., D.M.D., Professor of Operative Dentistry.
LUTHER D. SHEPARD, D.D.S., Adjunct Professor of Operative Dentistry.
NATHANIEL W. HAWES, Assistant Professor of Operative Dentistry.
EDWARD S. WOOD, M.D., Assistant Professor of Chemistry.
HENRY P. BOWDITCH, M.D., Assistant Professor of Physiology.
WILLIAM H. ROLLINS, D.M.D , Instructor in Dental Pathology.
CHARLES A. BRACKET, D.M.D., Instructor in Dental Therapeutics.
IRA A. SALMON, D.D.S., University Lecturer on Operative Dentistry.
CHARLES B. PORTER, M.D., Demonstrator of Practical Anatomy.
CHARLES WILSON, D.M.D., Demonstrator in Charge.
GEORGE F. GRANT, D.M.D., Demonstrator of Mechanical Dentistry.

The plan of study of this School has been radically changed. Instead of the usual oral examinations held at the end of the course, written examinations are had at the end of each year on the subjects pursued during the year, and instead of the customary repetition each year of the lectures and studies of the preceding year, the course is progressive over a period of two years, and the student is required to pass a satisfactory examination in the first year studies before being allowed to proceed to the next. At the end of the course a satisfactory examination must be passed in all the subjects.

The new year begins Sept. 27th, 1877, and ends the last Wednesday in June, 1878. It is divided into two equal terms, with a recess of one week at Christmas.

The regular examinations will be held in the following order, viz.: At the end of the first year, anatomy, including dissection, physiology and general chemistry. A certificate from the demonstrator of anatomy will be required of each student, that he has satisfactorily dissected the three parts of the body.

At the end of the second year, dental pathology, including a knowledge of gestation and diseases of women so far as they affect the mouth and throat, dental materia medica and therapeutics, oral surgery and surgical pathology, operative and mechanical dentistry. The examinations in operative and mechanical dentistry will include actual operations and the preparation of specimens of mechanical dentistry.

Every candidate must be twenty-one years of age, and of good moral character; he must give evidence of having studied medicine or dentistry three full years; he must have spent at least one continuous year at this school, have presented a satisfactory thesis and have passed all the required examinations.

The University Degree, D.M.D. (*Dentariæ Medicinæ Doctor*), is conferred upon those who fulfil the requirements.

Students may be admitted to advanced standing upon passing a satisfactory examination in a majority of the studies already pursued by the class; but no student shall advance with his class or be admitted to advanced standing until he has passed such examination.

The faculty recommend young men who propose to take the degree to spend the whole of the required term of three years of study in the school. But those who wish to spend but two of the three years in the school are earnestly advised to pass their first year of study, before entering, under the direction of a *competent* private instructor.

Unsurpassed facilities for operative and mechanical practice are furnished by the infirmary of the Massachusetts General Hospital which is open throughout the year.

**FEES.**

There are no fees for matriculation, for the diploma, nor for the demonstrators. For the first year a student is a member of the school, the fee is $200; for the second year, $150; for any subsequent year, $50.

For further information address,

**THOS. H. CHANDLER, Dean,**

**222 Tremont Street, Boston, Mass.**

Feb-1y.

*American Dental Association*—Meets on the first Tuesday in Aug., 1878, at Niagara Falls, *Pres.* F. H. Rehwinkel, Chillicothe, Ohio; *Cor. Sec.* J. H. McQuilien, Philadelphia; *Rec. Sec.* M. S. Dean, Chicago.

*American Dental Convention*—Meets at Niagara Falls, first Tuesday in August, 1878. *Pres.* J. H. Taft, Cincinnati, Ohio *Rec. Sec.* A. Tees, Philadelphia, Pa.

LIST OF SOCIETIES REPRESENTED IN THE AMERICAN DENTAL ASSOCIATION:

*American Academy of Dental Surgery*—Meets in New York 1878. *Pres.* Geo. H. Perine; *Rec. Sec.* N. M. Beckwith.

*American Academy of Dental Science*—Meets at Boston, Mass. *Pres.* Dr. D. M. Parker, *Sec.* W. L. Tucker, of Boston: *Cor. Sec.* Geo. T. Moffatt.

*Baltimore Dental Society*—*Pres.* C. T. Brockett; *Rec. Sec.* F. F. Drew.

*Brooklyn Dental Society*—Meets semi-monthly. *Pres.* H. G. Merick; *Rec. Sec.* C. P. Crandell.

*Buffalo Dental Society*—Meets second Monday of each month, at Buffalo. *Pres.* S. A. Freeman; *Rec. Sec.* G. C. Daboll.

*Boston Dental Institute*—Meets first Monday of each month. *Pres.* D. G. Williams, Boston; *Cor. Sec.* D. S. Dickerman.

*California State Dental Association*—Meets First Tuesday in June, 1878. *Pres.* Wm. B. Kingsbury; *Sec.* H. J. Plainteaux.

*Chicago Dental Society*—Meets monthly at Chicago. *Pres.* C. R. E. Koch; *Rec. Sec.* D. B. Freeman.

*Connecticut Valley Dental Association*—Meets semi-annually, second Tuesday of June and October. *Pres.* H. F. Bishop, worcester, Mass.; *Rec. Sec.* C. T. Stockwell, Westfield, Mass. Next meeting in Springfield, Mass., October 17, 1877.

*Connecticut State Dental Society*—*Pres.* E. T. Payne; *Rec. Sec.* Geo. L. Parmelee. Hartford. Semi-annual meeting in October in Hartford.

*Charleston Dental Association*—Meets second Tuesday of every month. *Pres.* T. F. Chupein; *Sec.* and *Treas.* B. A. Muckenfuss.

*Central Pennsylvania Dental Association*—Meets at Huntingdon, Jan.. 1878. *Pres.* E. J. Green; *Sec.* W. B. Miller.

*Cincinnati Dental Society*—Meets first and third Tuesday of each month. *Pres.* A. Berry; *Sec.* A. Downing.

*Central Ohio Dental Society*—Meets at Crestline, on the second Monday in April, 1877. *Pres.* M. DeCamp; *Sec.* N. J. Cressinger.

*Dental Society of the State of Maryland and District of Columbia*—Meets annually. Next meeting at Deer Park, on the second Tuesday in August, 1877. *Pres.* R. F. Hunt, of Washington, D. C.; *Sec.* H. C. Thompson.

*Eastern Indiana Dental Association*—Meets semi-annually, first Tuesday in May, and last Tuesday in October; *Pres.* J. W. Ellis, New Castle; *Sec.* M. H. Chappell, Knightstown, Ind. Place of meeting, Cambridge City.

*East Tennessee Dental Association*—Meets annually. Next meeting will be held at Cleveland, Tenn., on the third Wednesday of Sept. 1877. *Pres.* James Carson. *Sec.* J. U. Lee, of Chattanooga.

*Georgia State Dental Society*—July 30th, 1878. *Pres.* M. H. Thomas, Monroe Ga. *Rec. Sec.* J. A. Chopple, La Grange, Ga. *Cor. Sec.* Dr. M. S. Jobson, Perry, Ga.

*Harris Dental Association*—Meets quarterly. Next Meeting at Columbia, Pa., first Thursday in August. *Pres.* J. D. Heiges; *Sec.* W. N. Amer, Lancaster, Pa.

*Hudson Valley Dental Association*—Meets monthly at Troy, N. Y. *Pres.* L. C. Wheeler; *Rec. Sec.* S. G. Andres; *Cor. Sec.* S. D. French.

*Illinois State Dental Society*—Meets annually. *Pres.* C. R. E. Koch, Chicago; *Sec.* E. D. Swain, Chicago. Next place of meeting, Rockford, second Tuesday in May, 1878.

*Indiana State Dental Society*—Meets next year at Indianapolis, on the last Tuesday of June, 1878. *Pres.* Dr. T. S. Hacker, Ft. Wayne; *Sec.* J. E. Cravens.

*Iowa State Dental Society*—Meets annually. Next meeting at Iowa City on the second Tuesday of May. *Pres.* G. S. Shattuck, Belle Plane; *Rec. Sec.* E. E. Hughes of Newton; *Cor. Sec.* I. P. Wilson, Burlington.

*Kentucky State Dental Association*—Meets annually first Tuesday in June. Next meeting in Covington. *Pres.* A. H. Smith, of Richmond; *Sec.* F. Peabody of Louisville.

*Kansas State Dental Association*—*Pres.* J. D. Patterson; *Sec.* Wm. H. Shulze, of Atchison. Next meeting will be held at Lawreuce, first Tuesday in May, 1878.

*Lake Erie Dental Association*—Meets annually. *Pres.* C. B. Ansart, Oil City, Penn. *Sec.* C. D. Elliot, Franklin, Penn. Next meeting first Tuesday of May, 1876.

*Mad River Dental Association*—Meets at Xenia on the first Tuesday in April and October. *Pres.* R. Corson, Middletown; *Sec.* E. Betty, Xenia.

*Maine Dental Society*—Meets in August, at Thomaston. *Pres.* E. J. Roberts, Augusta, *Sec.* C. E. Hussey, Biddeford.

*Massachusetts Dental Society*—Meets monthly. *Pres.* John T. Codman, Boston; *Rec. Sec.* G. F. Grant, Boston; *Cor. Sec.* T. N. Chandler, Boston.

*Michigan State Dental Association*—Meets annually. Next meeting at Ann Arbor, October 9. *Pres.* R. G. Thomas, Detroit; *Sec.* and *Treas.* E. S. Holmes, Grand Rapids.

*Missippi Valley Dental Association*—Meets annually at Cincinnati, on first Wednesday in March, 1878. *Pres.* A. O. Rawls, Lexington, Ky.; *Rec. Sec.* Wm. Taft, Cincinnati.

*Mississippi State Dental Association*—Will meet third Wednesday of August, 1878, at Winona. *Pres.* A. H. Hilzheim, Jackson; *Sec.* M. C. Marshall.

*Minnesota Dental Association*—Meets annually. Meets May 26, 1878 at Winona. *Pres.* W. F. Lewis; *Sec.* C. M. Bailey, Minneapolis.

*Missouri Dental Association*—Meets annually on the first Tuesday of June. Next meeting at Sweet Springs. *Pres.* G. W. Tindall; *Sec.* W. H. Eames, of St. Louis,

*Missouri Valley Dental Association*—Meets on the fourth Tuesday in July, 1876, in Omaha, Neb. *Pres.* J. W. Chadduck; *Sec.* and *Treas.* F. M. Shrirer, Tabor, Iowa.

*Merrimac Valley Dental Association*—Meets on the 3d and 4th of May, 1878, at Lynn. *Pres.* C. W. Davis, New Bedford Mass.; *Rec. Sec.* W. E. Riggs, Lawrence, Mass.; *Cor. Sec.* D. W. Edgerly, Farmington, Mass.

*Memphis Dental Society*—Meets on the first Tuesday of each month. *Pres.* W. M. Arrington; *Sec.* V. F. Elliot; *Treas.* C. L. Harris.

*Nashville Dental Association*—Meets on the second Tuesday of each month. *Pres.* R. R. Freeman; *Sec.* L. G. Noel, Nashville.

*Nebraska State Dental Society*—Meets annually. Next meeting July 23, 1877, at Fremont. *Pres.* S. H. King. Lincoln. *Sec.* W. F. Roseman, Fremont.

*New Jersey State Dental Society*—Meets annually. Next meeting at Long Branch, on the second Tuesday in July, 1877. *Pres.* T. B. Welch, Newark; *Sec.* C. A. Meeker, Newark.

*New Haven Dental Society*—Meets on the first Wednesday of each month at New Haven. *Pres.* J. T. Metcalf; *Rec.* and *Cor. Sec.* C. L. Smith, of New Haven.

*New Orleans Dental Society*—Meets on the first Thursday of each month. *Pres.* W. S. Chandler; *Rec. Sec.* F. J. Knapp.

*Northern Ohio Dental Association*—Meets annually. Next meeting at Elyria, on the second Tuesday in May, 1878. *Pres.* J. W. Lyder, Akron, O. *Sec.* J. F. Siddall, Oberlin.

*Northern Iowa Dental Asssociation*—Meets annually, on the second Tuesday in June, 1873. Next meeting at waterloo. *Pres.* J. N. Abbott Lancaster; *Rec. Sec.* A. V. Eaton. Anamosa; *Cor. Sec.* A. R. Mason, Waterloo.

*North Carolina Dental Association*—Meets annually. Next meeting at Charlotte first Tuesday in Sept. 1878. *Pres.* J. W. Hunter, Salem; *Sec.* J. H. Crawford, Raleigh.

*North Carolina State Dental Society*—Meets annually. Next meeting at Raleigh, first Wednesday Sept. 1877. *Pres.* V. E. Turner; *Sec.* D. A. Robinson.

*Ohio College Dental Association*—Meets annually, February 25, at Cincinnati. *Pres.* N. G. McKellops, St. Louis, Mo.; *Rec. Sec.* H. A. Smith, of Cincinnati.

*Ohio State Dental Association*—Meets annually at Columbus, first Wednesday of December, 1877. *Pres.* I. Williams; *Rec. Sec.* H. L. Ambler.

*Odon ographic Society of Pennsylvania*—Meets on the first Wednesday of each month, at 8 o'clock, P.M., in the Philadelphia Dental College. *Pres.* F. M. Dixon; *Rec. Sec.* E. L. Hewitt.

*Odontological Society of New York City*—Meets the second Tuesday of each month. *Pres.* A. L. Northrup; *Rec. Sec.* Wm. Jarvie, Jr.

*Old Colony Dental Association*—Meets quarterly.* *Pres.* E. L. Hathaway; *Rec. Sec.* L. W. Puffer, North Bridgewater, Mass.

*Ohio Valley Dental Association*—Meets annually in N. E., Ky.; this year in Paris, Ky., Tuesday, September 9th.

*Oregon State Dental Society*—Meets annually. *Pres.* J. R. Cardwell, Portland; *Rec. Sec.* G. N. Chance, Portland.

*Pennsylvania State Dental Society*—Meets next in Minnequa Springs, second Tuesday of July, 1877. *Pres.* J. S. King; *Rec. Sec.* S. H. Guilford; *Cor Sec.* M. H. Webb.

*Pennsylvania Association of Dental Surgeons*—Meets monthly in Philadelphia. *Pres.* Robert Henry; *Sec.* G. Pettit.

*Pittsburg Dental Society*—Meets second Tuesday of each month. *Pres.* H. W. Arthur, of Alleghaney; *Sec.* E. C. Diehl, Pitttbuagh.

*San Francisco Dental Association*—Meets monthly. *Pres.* Alex. Warner; *Vice Pres.* R. F. Rall; *Cor. Sec.* S. E. Goe; *Rec. Sec.* L. L. Dunbar.

*St. Louis Dental Society*—Meets monthly. *Pres.* W. H. Morrison; *Sec.* A. H. Fuller.

*Susquehanna Dental Association*—Meets annually. *Pres.* J. N. Johnston; *Rec. Sec.* E. B. Long. Meets November 14, 1877, at Wilkesbarre.

*Society of Dental Surgeons of the City of New York*—Meets semi-monthly in New York. *Pres.* B. F. Franklin; *Rec.* J. S. Latimer; *Cor. Sec.* E. H. Burras.

**Southern Dental Association*—Meets annually. Next meeting at Deer Park, August 14. 1877. *Pre.* J. S. Cobb, Nashville, Tenn.; *Rec. Sec.* E. E. Chisholm, Tuscaloosa, Ala.

*South Carolina State Dental Assocation*—Meets annually. *Pres.* G. F. S. Wright, Columbia; *Sec.* T. F. Cheupein, Charleston; *Cor. Sec* J. S. Thompson, Abbeville. Next meeting at Columbia, the first Tuesday of June, 1877.

*Tennessee Dental Association*—Meets semi-annually. Next meeting at Lookout Mountain June 27, 1877. *Pres.* H. E. Beach; *Sec.* E. S. Chisholm, Tuscaloosa, Ala., *Rec. Sec.* W. H. Morgan; *Cor. Sec.* L. G. Noel.

*Virginia State Dental Association*—Meets in Richmond December 14. *Pres.* W. W. H. Thackston, Farmville; *Sec.* G. F. Keesee, Richmond.

*Washington City Dental Society*—Meets monthly. *Pres.* J. C. Smith; *See.* J. B. TenEyck.

*Wisconsin State Dental Association*—Meets annually. Next meeting at Milwaukee, second Tuesday of July, 1877. *Pres.* R. S. Wells, Sparta; *Sec.* M. T. Moore, La Crosse.

### State and District Dental Societies of State of New York.

*New York State Dental Society*—Meets at Albany on the second Wednesday of July next *Pres.* W. C. Barrett, Warsaw, N. Y.; *Cor. Sec.* S. B. Palmer; *Rec. Sec.* S. S. A. Freeman, Buffalo.

*First District*—Meets second and fourth Wednesday evenings of each month. Annual meeting in New York, first Tuesday in June. *Pres.* J. S. Latimer, New York; *Rec. Sec.* F. M. Odell, New York.

*Second District*—Annual meeting in Brooklyn, first Monday in April. *Pres.* C. D. Cook; *Rec. Sec.* G. L. Mason, Brooklyn; *Cor. Sec.* W. S. Elliott. The meetings are held quarterly, second Thursday in January, April, July and October.

*Third District*—Meets semi-annually on second Tuesday in January and July. Annual meeting at Albany on second Tuesday in January. *Pres.* S. P. Welsh; *Rec. Sec.* H. A. Hall, Troy; *Cor. Sec.* J. A. Perkins, Albany.

*Fourth District*—Meets semi-annually. Meets annually at Saratoga on second Tuesday in June. *Pres.* C. A. Elmore, Ft. Edward; *Rec. Sec.* E. S. Pearsall, Saratoga.

*Fifth District*—Meets semi-annually. Annual meeting at Syracuse, second Tuesday in June. *Pres.* F. D. Nellis, Syracuse; *Rec. Sec.* J. S. Marshall, Syracuse; *Cor. Sec.* A. B. Cowles, Rome.

*Sixth District*—Meets semi-annually. Aunual meeting at Binghampton, on second Tuesday in June. *Pres.* Frank B. Darby, Oswego; *Rec. Sec.* F. E. Perry, Norwich *Cor. Sec.* A. M. Holmes, Morrisville.

*Seventh District*—Meets semi-annually. Annual meeting at Rochester, first Tuesday in June. *Pres.* H. S. Miller; *Rec. Sec.* J. E. Line, Rochester.

*Eighth District*—Meets semi-annually. Annual meeting on second Tuesday in May. *Pres.* G. C. Daboll; *Rec. Sec.* S. A. Freeman; *Cor. Sec.* T. G. Lewis, Buffalo.

*The Sevent and Eighth District Societies*—Hold a union meeting on the last Tuesday of October, alternately at Buffalo and Rochester.

*The Ohio State Board of Dental Examiners* will meet in Columbus on the first Tuesday of December, 1877, at 12 o'clock, M. *Pres.* J. Taft; *Sec.* W. P. Horton.

### EXCHANGES.

ST. LOUIS MEDICAL AND SURGICAL JOURNAL.
BOSTON MEDICAL AND SURGICAL JOURNAL.
THE PACIFIC MEDICAL AND SURGICAL JOURNAL, SAN FRANCISCO.
PHILADELPHIA MEDICAL AND SULGICAL JNURNAL.
CINCINNATI MEDICAL ADVANCE.
CINCINNATI LANCET AND OBSERVER.
DENTAL COSMOS, PHILADELPHIA.
LADIES' REPOSITORY, CINCINNATI.
CHICAGO MEDICAL EXAMINER.
THE DETROIT REVIEW OF MEDICINE AND PHARMACY.
MEDICAL RECORD, N. Y.
NASHVILLE JOURNAL OF MEDICINE AND SURGERY.
AMERICAN JOURNAL OF DENTAL SCIENCE, BALTIMORE.
THE UNITED STATES MEDICAL AND SURGICAL JOURNAL, CHICAGO.
BOSTON JOURNAL OF CHEMISTRY.
JOURNAL OF APPLIED CHEMISTRY.
CANADA JOURNAL OF DENTAL SCIENCE, MONTREAL.
INDIANA JOURNAL OF MEDICINE.
MEDICAL AND SURGICAL REPORTER, SALEM, OREGON.
MISSOURI DENTAL JOURNAL. ST. LOUIS.
ECLECTIC MEDICAL JOURNAL, CINCINNATI.
THE SANITARAN, NEW YORK CITY.

### HARVARD UNIVERSITY.

### Dental Department.

### BOSTON, MASS. 1877-87.

**FACULTY.**

A MONTHLY MAGAZINE DEVOTED TO THE INTERESTS OF

**THE DENTAL PROFESSION.**

*TERMS REDUCED TO $2.50 PER YEAR. SINGLE COPIES 25C.*

EDITED BY PROF. J. TAFT, D.D.S.

AND

*Published by SPENCER, CROCKER & CO., Ohio Dental Depot.*

The volume commences in January and closes in December.

The REGISTER being issued monthly is always fresh, therefore Indispensable to the practitioner who desires to keep posted up with the new inventions and improvements which are daily developed. Neither expense nor effort will be spared to give value and variety to its contents. Articles will be illustrated with well executed engravings whenever they will add to the interest or assist to explanation.

Its extensive circulation and frequency of issue make it one of the BEST DENTAL ADVERTISING MEDIUMS in the United States.

All subscriptions must begin with January or July.

Local or special notices, five lines or less, will be inserted for $[illegible] each insertion; over five lines, 50 cts. per line.

All advertising bills payable in advance, or at furthest, after the issue of the first number containing the advertisement. All transient advertisements to be paid for in advance.

☞CONTRIBUTIONS SOLICITED.

Exclusively Editorial Communications should be addressed to Editor.

The latest number of the REGISTER may be ordered with or without other goods at any time, and all letters should be addressed to

*SPENCER, CROCKER & CO., Ohio Dental Depot, Cincinnati, O.*

